# Trifunctional nanoprecipitates ductilize and toughen a strong laminated metastable titanium alloy

**DOI:** 10.1038/s41467-023-37155-y

**Published:** 2023-03-13

**Authors:** Chongle Zhang, Shuaiyang Liu, Jinyu Zhang, Dongdong Zhang, Jie Kuang, Xiangyun Bao, Gang Liu, Jun Sun

**Affiliations:** State Key Laboratory for Mechanical Behavior of Materials, Xi’an Jiaotong University, Xi’an, People’s Republic of China

**Keywords:** Metals and alloys, Mechanical properties

## Abstract

Metastability-engineering, e.g., transformation-induced plasticity (TRIP), can enhance the ductility of alloys, however it often comes at the expense of relatively low yield strength. Here, using a metastable Ti-1Al-8.5Mo-2.8Cr-2.7Zr (wt.%) alloy as a model material, we fabricate a heterogeneous laminated structure decorated by multiple-morphological α-nanoprecipitates. The hard α nanoprecipitate in our alloy acts not only as a strengthener to the material, but also as a local stress raiser to activate TRIP in the soft matrix for great uniform elongation and as a promoter to trigger interfacial delamination toughening for superior fracture resistance. By elaborately manipulating the activation sequence of lamellar-thickness-dependent deformation mechanisms in Ti-1Al-8.5Mo-2.8Cr-2.7Zr alloys, the yield strength of the present submicron-laminated alloy is twice that of equiaxed-coarse grained alloys with the same composition, yet without sacrificing the large uniform elongation. The desired mechanical properties enabled by this strategy combining the laminated metastable structure and trifunctional nanoprecipitates provide new insights into designing ultra-strong and ductile materials with great toughness.

## Abstract (web summary)

Engineering metastability can enhance the ductility of alloys, but it often comes at an expense of relatively low yield strength. Here, the authors fabricate a Ti-based alloy with heterogeneous laminated structures and α-nanoprecipitates that enhance yield strength without sacrificing ductility.

## Introduction

Materials possessing superior strength and excellent ductility simultaneously have always been in high demand; unfortunately, these properties are generally mutually exclusive, which is referred to as the strength-ductility trade-off dilemma^1–8^. Transformation/twinning-induced plasticity (TRIP/TWIP) mechanisms confer these metastable alloys, such as conventional steels^6^ and Ti alloys^7,9–14^ and recently emerging multicomponent alloys^15–17^, enhanced work-hardening rates (*θ* > 2000 MPa) and good ductility to balance the conflict between strength and ductility, whereas they often manifest very low yield strength (*σ*_y_). Given that the strength *σ*_y_ is one of the most important characteristics of structural materials^18^, it is natural to inquire whether the TRIP/TWIP mechanisms, e.g., stress-induced martensite (SIM) can be replaced by the ordinary dislocation plasticity (ODP) that is usually activated at high stresses and subsequently TRIP/TWIP switches on to achieve large ductility (ɛ_f_), particularly uniform elongation (ɛ_U_), thus enhancing the fracture resistance of these alloys.

As a matter of fact, there are two classic strategies to strengthen metastable alloys. The first is to tailor the deformation mechanisms transformed from SIM to twinning, and even to ODP by increasing the phase stability^9,11,19^. However, this strategy failed to tune the sequential activation of mechanisms from ODP to SIM or twinning in a metastable phase without variation in the chemical composition; namely, there is no deformation mechanism transition with increasing plastic strains when ODP occurs first in alloys. The second is to architect heterogeneous structures in terms of the spatial confinement imposed by grain boundaries (GBs), layer interfaces, and/or precipitates to control the activation of plastic carriers^20–23^, among which the heterogeneous laminate structure (HLS) enables us to readily achieve this goal via the combination of ODP and SIM in hard-stable and soft-metastable constituents, respectively^6,24^. Thus, it is a great challenge by far to realize the sequential activation from ODP to SIM or twinning with increasing stresses/strains through the size-constraining effect that can effectively delay the activation of TRIP or TWIP in a single-phase metastable alloy. It is desirable, thus, to devise a heterogeneous structure that can fully utilize the size-dependent mechanism transition with strains in a metastable phase, to activate ODP first and then SIM or twinning to notably enhance *σ*_y_ without sacrificing ɛ_f_ (especially ɛ_U_).

In this work, unlike the prior HLS with a high fraction of the soft-metastable phase in the hard matrix^6,23^, we propose a different design concept—namely, a low fraction of multi-morphologically hard-yet-deformable α nanoprecipitates distributed in the soft laminated metastable β-matrix to achieve a desired combination of strength and ductility in a model Ti-1Al-8.5Mo-2.8Cr-2.7Zr (wt%) alloy. Based on the “*d*-electron design method”^19,25,26^, we design this β-Ti alloy (see Supplementary Note 1 and Supplementary Figs. 1–3). The β-phase stability was tailored via elemental partitioning by producing α nanoprecipitates to trigger SIM (after ODP) at very high stresses by lamination, without altering the global chemical composition of Ti alloys (the detailed processing procedure can be found in the Methods, Supplementary Fig. 4 and Supplementary Table 1). On the one hand, these α nanoprecipitates serve as strong pinning particles to hinder β-GB migration and dislocation motion to strengthen alloys; on the other hand, they serve as local stress raisers to stimulate SIM in the β-matrix and subsequently trigger interfacial delamination toughening to ductilize/toughen alloys. As a result, compared with their equiaxial-grained structure (EGS) siblings, the yield strength *σ*_y_ of HLS alloys decorated with the trifunctional α nanoprecipitates is doubled almost without losing their uniform elongation ɛ_U_ utilizing the size-dependent deformation mechanism transition from SIM to ODP. Our findings demonstrate how deformation mechanisms can be deliberately activated by tailoring the characteristic size (e.g., grain sizes and precipitate spacing) of the microstructure, along with the controllable phase stability of the matrix, to optimize strength and ductility for superior fracture resistance.

## Results

### Hierarchical microstructure and deformation mechanisms of the HLS Ti alloys

**Fig. 1:**
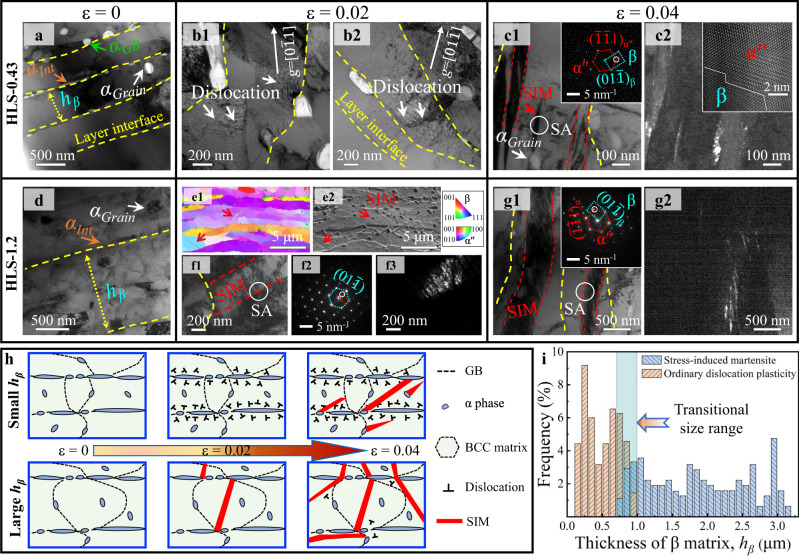
Deformation mechanisms in HLS-0.43 and HLS-1.2 β-Ti alloys activated in the initial plastic deformation stage. **a**–**c** HLS-0.43 β-Ti alloys. **a** At a strain ɛ = 0, a bright-field TEM (BF-TEM) image shows the layered structure and multiple phases. **b1-b2** At a strain ɛ = 0.02, dislocations are generated from the α/β interface and pile up against the opposite interface, and no SIM is observed in the β layers. **c1** At a strain ɛ = 0.04, a BF-TEM image shows SIM bands are nucleated from the α/β interface and in a region adjacent to α_Grain_ inside the β-layer, indicated by red dashed lines and red arrow, respectively, which are verified by the SAED pattern in **c1**. **c2** The DF-TEM image of **c1**. The inset is the corresponding HR-TEM image in **c2**, showing the α″/β interface highlighted by the white line. **d**–**g** HLS-1.2 β-Ti alloys. **d** A BF-TEM image of upstretched HLS-0.43 β-Ti alloys. **e1-e2** The inverse pole figure (IPF) and corresponding SEM maps show the activation of SIM. **f1** A typical DF-TEM image shows SIM initiated from the α/β interface, as verified by **f1** the SAED pattern and **f3** the DF-TEM image of SIM. **g1** At a strain ɛ = 0.04, a BF-TEM image shows two SIM bands was activated, indicated by red dashed lines, as verified by the SAED pattern in **g1** and **g2** the DF-TEM image. **h** Schematic illustration of the deformation mechanism evolution of two different β-layer thickness samples during deformation. **i** Histogram showing the activation of deformation mechanisms, i.e., ODP and SIM, in β-layers at a strain ɛ = 0.02. Beam parallel to a <011>_β_ zone axis in **c1** and **g1**. Beam parallel to a < 11$\bar{1}$ >_β_ zone axis in **f2**.

The partially recrystallized β-Ti alloys that we explored to investigate the validity of our concept exhibit an intrinsic HLS with metastable β-layers decorated with interfacial α (*α*_Int_), intragranular α (*α*_Grain_) and intergranular α (*α*_GB_) nanoprecipitates, as shown in Fig. 1a. More specifically, in the layer thickness (*h*_*β*_) direction, almost only one submicron-sized elongated β-grain is aligned with the rolling direction, i.e., the β-layer is almost composed of single-layered grains (also see Supplementary Figs. 5–7). Thus, the present HLS β-Ti alloys possess the key combination of three characteristics, i.e., multiple phases, metastability, and lamination. More details about the statistical results for the sizes of microstructural features are displayed in Supplementary Figs. 5–9 and Supplementary Tables 2, 3. To illustrate the deformation mechanisms of our Ti-Al-Mo-Cr-Zr HLS alloys, their microstructural evolution with the plastic strain (ɛ) should be uncovered. For the HLS β-Ti alloy with *h*_*β*_ = 0.34 μm (termed as the HLS-0.34 sample hereafter), only ODP was switched on, implying the suppression of SIM at such a small size during plastic deformation. Interestingly, dislocations pile up against the α/β interfaces in lieu of SIM that emerged in the HLS-0.43 alloy at ɛ = 0.02, as shown in Fig. 1b1-b2. Beyond this strain, apart from the SIM nucleated from α/β hetero-phase interfaces, some β-regions adjacent to α precipitates transformed into the orthorhombic α″ martensitic structure (i.e., SIM), as marked by the red arrow in Fig. 1c1. In contrast, for the ɛ = 0.02 HLS-1.2 β-Ti alloy, SIM initiated from the α/β interface/boundary was activated in thick rather than thin β-layers, see Fig. 1e, f. This phase transformation was verified by the corresponding selected area electron diffraction (SAED) and dark-field transmission electron microscopy (DF-TEM) images in Fig. 1f2 and f3, respectively. At ɛ = 0.04, wider and longer SIM plates were activated to accommodate plastic deformation in the HLS-1.2 sample, see Fig. 1g1-g2. These findings imply that there is a strong *h*_*β*_-size effect on SIM in HLS Ti alloys. Thus, we determined the critical size for the SIM to ODP transition in ɛ = 0.02 stretched HLS samples from a large number (316) of β-layers (including 178 β-layers from TEM images and 138 β-layers from electron backscatter diffraction (EBSD) images) by identifying the deformation products, as shown in Fig. 1i. Apparently, ODP only operates in β-layers with *h*_*β*_ < 0.7 µm, while SIM only operates in β-layers with *h*_*β*_ > 1.0 µm. In the transition *h*_*β*_-regime (0.7–1 µm), both ODP and SIM switch on, but SIM becomes more difficult with reducing *h*_*β*_.

### Homogenous microstructure and deformation mechanisms of EGS Ti alloys

**Fig. 2:**
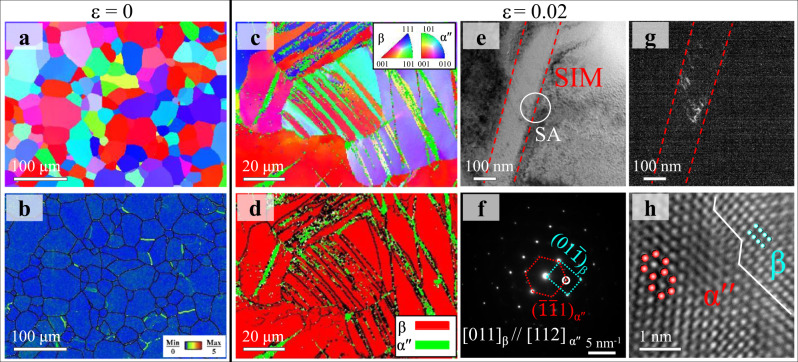
Deformation mechanisms of the EGS-61 β-Ti alloy at the initial stage of plastic deformation. At a strain ɛ = 0, **a** the inverse pole figure (IPF) image and **b** the corresponding kernel average misorientation (KAM) image. **c** At a strain ɛ = 0.02, the IPF image shows the plate-like deformation bands that were identified to be SIM α″, as verified by **d** the corresponding α″ phase map. **e** A typical BF-TEM image shows a SIM band with a width ∼of 150 nm activated in the deformed EGS-61 sample, as verified by **f** the SAED pattern and **g** the DF-TEM image of SIM. **h** A typical HR-TEM image shows the β/α″ interface, as marked by the white line.

Figure 2 shows the microstructural features of the present EGS β-Ti alloy with an average grain size *d* ~61 μm (termed as the EGS-61 sample hereafter). The as-rolled β-Ti alloys after a long solution treatment time exhibited the fully recrystallized coarse-grained EGS feature (Fig. 2a) with a very low dislocation density (see Fig. 2b and Supplementary Fig. 10). To clarify the activation of TRIP and the evolution of microstructure in EGS-61 alloys, EBSD mapping was performed in the early deformation stage. In the ɛ = 0.02 stretched EGS-61 sample, SIM was first activated during tension, consistent with previous studies^27,28^. Figure 2c shows that plate-like deformation bands with different orientations (coded by the color contrast) were activated in β grains and identified to be SIM α″, as verified by the corresponding α″ phase map in Fig. 2d and the TEM observations in Fig. 2e–g. The β/α″ phase interface shows a classical Burgers orientation relationship^29^ of ${[011]}_{\beta }$//${[112]}_{\alpha {\prime\prime} }$, as indicated by the white line in Fig. 2h.

### Mechanical properties of HLS Ti alloys

**Fig. 3:**
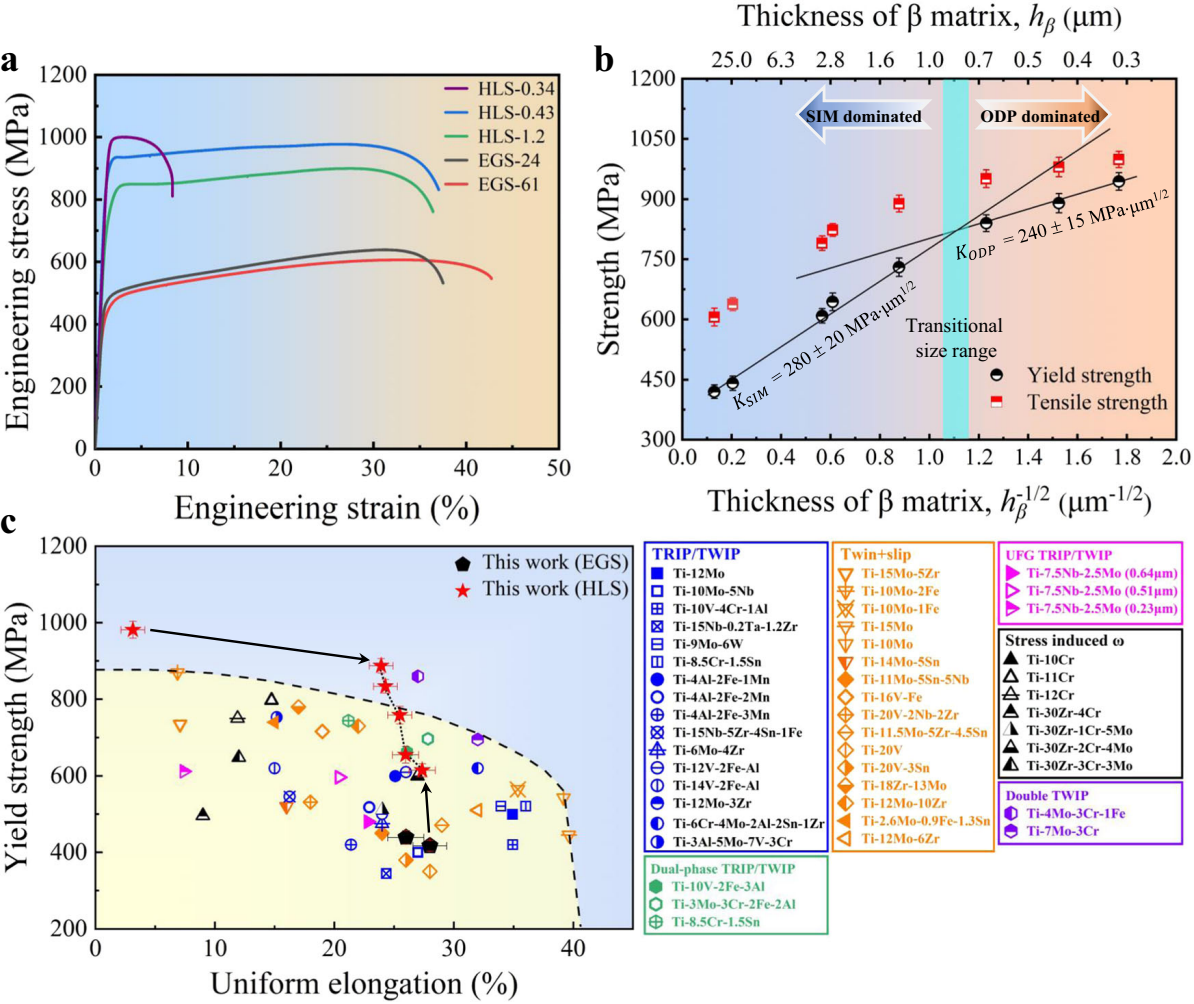
The mechanical responses of β-Ti alloys. **a** The engineering stress–strain curves. **b** The yield/tensile strength of the present β-Ti alloys with different β-layer thicknesses. The predicted critical stress for the activation of SIM and ODP is associated with different H–P slopes, i.e., ${K}_{{{{{{{\mathrm{SIM}}}}}}}}$ = 280 ± 20 MPa μm^1/2^ for SIM and ${K}_{{{{{{{\mathrm{ODP}}}}}}}}$ = 240 ± 15 MPa μm^1/2^ for ODP, revealing a critical size of ~0.80 ± 0.07 μm for the SIM to ODP transition. **c** A comparison of yield strength *vs*. uniform elongation of our β-Ti alloys with reported metastable β-Ti alloys, including TRIP/TWIP Ti alloys: Ti-12Mo^9,54^, Ti-10Mo-5Nb^43^, Ti-10V-4Cr-1Al^10^, Ti-15Nb-0.2Ta-1.2Zr^20^, Ti-9Mo-6W^26^, Ti-8.5Cr-1.5Sn^37^, (Ti-4Al-2Fe-1Mn, Ti-4Al-2Fe-2Mn and Ti-4Al-2Fe-3Mn)^38^, Ti-15Nb-5Zr-4Sn-1Fe^39^, Ti-6Mo-4Zr^51^, (Ti-12V-2Fe-1Al and Ti-14V-2Fe-1Al)^44^, and Ti-12Mo-3Zr;^50^ Dual-phase TRIP/TWIP Ti alloys: Ti-10V-2Fe-3Al^13^, Ti-3Mo-3Cr-2Fe-2Al^42^, Ti-8.5Cr-1.2Sn;^14^ Twin+slip Ti alloys: Ti-3Al-5Mo-7V-3Cr^35^, (Ti-15Mo-5Zr, Ti-10Mo-2Fe, Ti-10Mo-1Fe and Ti-15Mo)^36^, Ti-10Mo, (Ti-14Mo-5Sn and Ti-11Mo-5Sn-5Nb)^43^, Ti-16V-1Fe^44^, Ti-20V-2Nb-2Zr^45^, (Ti-11.5Mo-5Zr-4.5Sn, Ti-20V-3Sn and Ti-20V)^46^, Ti-6Cr-4Mo-2Al-2Sn-1Zr^47^, Ti-18Zr-13Mo^48^, (Ti-12Mo-10Zr and Ti-12Mo-6Zr)^50^, Ti-2.6Mo-0.9Fe-1.3Sn;^49^ UFG TRIP/TWIP Ti alloys: Ti-7.5Nb-2.5Mo (with different β grain sizes);^41^ Stress-induced ω Ti alloys: (Ti-10Cr, Ti-11Cr, and Ti-12Cr)^64^, (Ti-30Zr-4Cr, Ti-30Zr-1Cr-5Mo, Ti-30Zr-2Cr-4Mo and Ti-30Zr-3Cr-3Mo);^40^ Double TWIP Ti alloys: Ti-7Mo-3Cr^52^ and Ti-4Mo-3Cr-1Fe^53^ alloys. Error bars indicate standard deviations for three tests. More details (alloy composition and corresponding references) can be found in Supplementary Table 5.

Figure 3a shows the engineering stress–strain curves of some HLS and EGS β-Ti alloys. Compared with EGS samples, HLS β-Ti alloys manifest notably increased flow stresses with decreasing *h*_*β*_, which is expected because the interfaces can block dislocation motion^30^. Although the critical stress of TRIP is often much lower than that of ODP (as well as TWIP) in metastable β-Ti alloys, there is no stress drop in the plastic flow of our transformable β-Ti alloys. It is suggested that the lamination constraining effects notably enhance the critical stress of SIM in our HLS alloys. The mechanical performance of alloys can be defined by some properties. The yield strength *σ*_y_ sets the resistance to the onset of plastic deformation. The uniform elongation ε_U_ (termed hereafter as the true strain, and determined based on Considère’s criterion, see Supplementary Fig. 10 and Supplementary Table 4) quantifies the resistance to plastic localization or necking and is directly related to the work-hardening capacity, with *σ*_UTS_ being the corresponding stress, i.e., ultimate tensile strength. The strength *σ*_y_ of HLS-0.43 β-Ti alloys is as high as ~890 MPa, which is more than twice that (~417 MPa) of their EGS-61 counterparts (see the inset in Fig. 3a), almost without sacrificing the uniform elongation ε_U_ ~23% in larger *h*_*β*_ HLS alloys. However, the HLS-0.34 samples have the highest *σ*_y_ ~953 MPa but the lowest ε_U_ ~3%. This demonstrates that the lamellar structure can be optimized to produce a desirable strength-ductility balance that is not accessible to their homogeneous counterparts.

Figure 3b shows both the yield strength and the tensile strength scale with the inverse square root of layer thickness *h*_*β*_, i.e., *h*_*β*_^−1/2^. Similar trends were observed for the yield strength vs. inverse β-layer thickness, see Supplementary Fig. 12. As mentioned earlier, in the large *h*_*β*_-regime, SIM is the dominant deformation mechanism, as in coarse-grained EGS samples. The strength ${\sigma }_{y}$ with respect to *h*_*β*_ clearly follows the Hall–Petch (H–P) relationship^31–33^ with the exponent of -1/2 and the slope ${K}_{{{{{{{\mathrm{SIM}}}}}}}}$ = 280 ± 20 MPa μm^1/2^ (Fig. 3b). In the small *h*_*β*_-regime, by contrast, SIM begins to give way to ODP at small stress/strain levels, however, the strength ${\sigma }_{y}$ still obeys the H–P relationship but with the low slope of ${K}_{{{{{{{\mathrm{ODP}}}}}}}}$ = 240 ± 15 MPa μm^1/2^. The SIM with a larger H–P slope manifests higher strengthening rates than ODP in the present β-Ti alloys, leading to a critical size of ~0.80 ± 0.07 μm for the deformation mechanism transition, quite approaching the statistical result of ~0.7–1 µm in Fig. 1i. Therefore, it seems that there is a strong size-constraining effect on the deformation mechanism transition from a highly coherent inelastic shearing process, i.e., SIM to the randomly dispersed inelastic shear activities among slip planes, i.e., ODP with a reduction in characteristic sizes in our designed alloys, similar to previous studies^34^. It should be noted that the tensile strength of the HLS-0.43 β-Ti alloys is almost equal to the predicted critical stress of stress-induced martensite, while the tensile strength of the HLS-0.34 samples is far below this predicted critical value. These findings imply that SIM still takes place in HLS-0.43 β-Ti alloys at high stresses but fully shuts down in HLS-0.34 samples, consistent with our observations above.

Figure 3c plots *σ*_y_ vs. ε_U_ of our β-Ti alloys and the reported metastable β-Ti alloys^9,10,13,14,20,35–54^. Compared with reported metastable β-Ti alloys by far, the HLS-0.34 β-Ti alloys show the highest *σ*_y_, while the HLS-0.43 β-Ti alloys show a desired combination of *σ*_y_ ~890 MPa and ε_U_ ~23%, comparable to the Ti-4Mo-3Cr-1Fe alloy^53^ (with *σ*_y_ ~870 MPa and *σ*_UTS_ ~1092 MPa) deformed via the activation of both {332}_*β*_ <113>_*β*_ and {112}_*β*_ <111>_*β*_ twinning systems. Specifically, the ductility ε_U_ ~23% of our HLS-0.43 β-Ti alloys is far larger than that of reported ultrafine-grained TRIP β-Ti alloys (e.g., *d* ~0.23 μm alloys with *σ*_y_ ~610 MPa and ε_U_ ~7%)^41^. It implies that our HLS β-Ti alloys (except the HLS-0.34 sample), utilizing the size-dependent deformation mechanism to trigger SIM (after ODP) at very high stresses, unite ultrafine-grain strength with coarse-grain ductility.

**Fig. 4:**
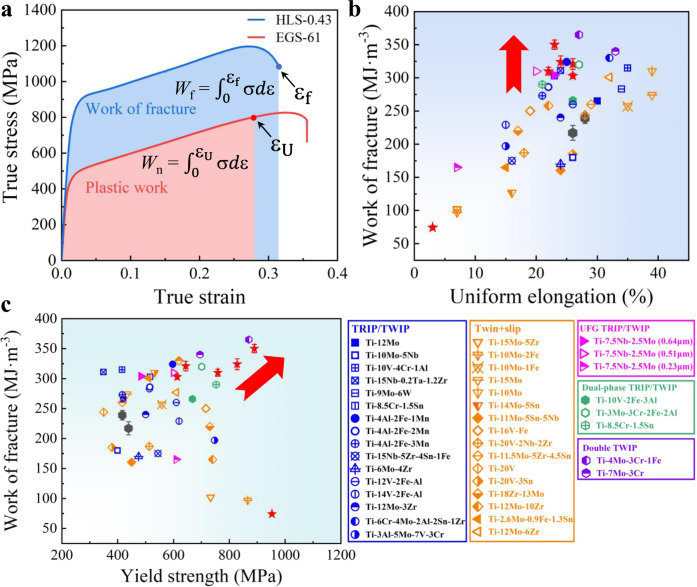
The fracture properties of β-Ti alloys. **a** The true stress–strain curves of HLS-0.43 and EGS-61 β-Ti alloys. The red and blue colored areas represent the integration performed to estimate the plastic work and work of fracture, respectively. Comparisons of **b** the work of fracture vs. true uniform strain and **c** the work of fracture vs. yield strength of our β-Ti alloys with reported metastable β-Ti alloys, including TRIP/TWIP Ti alloys: Ti-12Mo^9,54^, Ti-10Mo-5Nb^43^, Ti-10V-4Cr-1Al^10^, Ti-15Nb-0.2Ta-1.2Zr^20^, Ti-9Mo-6W^26^, Ti-8.5Cr-1.5Sn^37^, (Ti-4Al-2Fe-1Mn, Ti-4Al-2Fe-2Mn and Ti-4Al-2Fe-3Mn)^38^, Ti-15Nb-5Zr-4Sn-1Fe^39^, Ti-6Mo-4Zr^51^, (Ti-12V-2Fe-1Al and Ti-14V-2Fe-1Al)^44^, Ti-12Mo-3Zr^50^ and Ti-6Cr-4Mo-2Al-2Sn-1Zr;^47^ Dual-phase TRIP/TWIP Ti alloys: Ti-10V-2Fe-3Al^13^, Ti-3Mo-3Cr-2Fe-2Al^42^, Ti-8.5Cr-1.2Sn;^14^ Twin+slip Ti alloys: Ti-3Al-5Mo-7V-3Cr^35^, (Ti-15Mo-5Zr, Ti-10Mo-2Fe, Ti-10Mo-1Fe and Ti-15Mo)^36^, Ti-10Mo, (Ti-14Mo-5Sn and Ti-11Mo-5Sn-5Nb)^43^, Ti-16V-1Fe^44^, Ti-20V-2Nb-2Zr^45^, (Ti-11.5Mo-5Zr-4.5Sn, Ti-20V-3Sn and Ti-20V)^46^, Ti-18Zr-13Mo^48^, (Ti-12Mo-10Zr and Ti-12Mo-6Zr)^50^, Ti-2.6Mo-0.9Fe-1.3Sn^49^, UFG TRIP/TWIP Ti alloys: Ti-7.5Nb-2.5Mo (with different β grain sizes);^41^ Double TWIP Ti alloys: Ti-7Mo-3Cr^52^ and Ti-4Mo-3Cr-1Fe^53^ alloys. Error bars indicate standard deviations for three tests.

It is well accepted that the combination of strength and ductility determines the toughness and fracture resistance of structural materials, often represented by the work of fracture or the plastic work density (see Fig. 4a)^7,55^. In particular, the work of fracture (or static toughness) *W*_f_ = ${\int }_{0}^{{{{{{{\rm{\varepsilon }}}}}}}_{{{{{{\rm{f}}}}}}}}{{{{{\rm{\sigma }}}}}}d{{{{{\rm{\varepsilon }}}}}}$, characterizes the work per unit volume dissipated until final fracture in the fracture zone, which is closely related to the true fracture strain ε_f_ and the true fracture stress *σ*_f_ (see Fig. 4a). To evaluate the fracture resistance of the present HLS samples, we compare *W*_f_ vs. ε_U_ and *W*_f_ vs. *σ*_y_ for our alloys and other reported metastable β-Ti alloys, as shown in Fig. 4b, c, respectively. Figure 4b combines two indicators that determine the resistance to failure of ductile alloys: *W*_f_ for damage-controlled fracture and ε_U_ for plastic localization-controlled fracture. Note that except for the double twinned Ti-4Mo-3Cr-1Fe alloy, our (HLS-0.43) alloys have moderate ε_U_ but the highest *W*_f_ (and plastic work density *W*_n_, see Supplementary Fig. 13), originating from the desired combination of strength and ductility in our HLS alloys. Especially, the HLS-0.43 sample has a much larger *W*_f_ than that of the great damage-resistant Ti-12Mo alloy^7^, and its *σ*_y_ is about twice that of the Ti-12Mo alloy, see Fig. 4c.

**Fig. 5:**
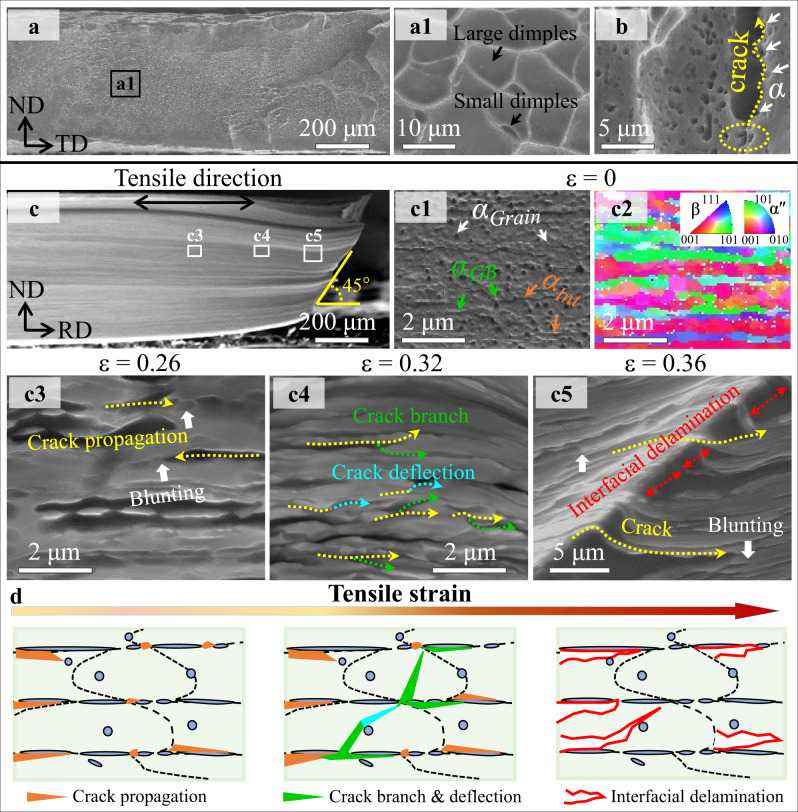
Fracture behavior and the underlying mechanism of HLS-0.43 β-Ti alloys. **a** An SEM image of the fracture surface of HLS-0.43 β-Ti alloys. **a1** A magnified image of the fracture surface shows massive dimples. **b** An SEM image shows that these cracks are initially nucleated at α/β layer interfaces. **c** An SEM image of the post-fractured HLS-0.43 sample along rolling direction. **c1-c2** The SEM and corresponding EBSD images show the configurations of undeformed α nanoprecipitates at ε = 0. **c3** The SEM images show crack initiation and propagation along the α/β interfaces at ε = 0.26, and cracks were blunted via α_Grain_ nanoprecipitates (white arrows), **c4** crack deflection (cyan arrows) and branch (green arrows) at ε = 0.32, and **c5** interface delamination at ε = 0.36. **d** Schematic illustration of the fracture process of HLS-0.43 β-Ti alloys during post-uniform elongation in terms of crack deflection and branch and interfacial delamination caused by α nanoprecipitates.

Compared with the fracture process of our EGS alloys without and with α-nanoprecipitates (see Supplementary Figs. 14, 15 in Supplementary Note 6), these transformable HLS samples (with *h*_*β*_ > 0.34 μm) exhibit unique fracture behavior. Using the HLS-0.43 sample as an example, we illustrate the exceptional ductility of our HLS alloys and their superior fracture resistance in light of the fracture process. For the present HLS-0.43 alloy, we characterized the fractography of the rupture surface, which shows obvious dimples as direct evidence of ductile fracture^7^; see Fig. 5a1. The corresponding fractography of the subsurface shows a crack initiates at the α/β interface (indicated by the yellow circle) and its propagation path is deflected by these α precipitates, as indicated by white arrows in Fig. 5b.

Figure 5c presents the fracture process in the HLS-0.43 β-Ti alloy by observing microcrack nucleation/evolution at different strains, revealing the fracture angle is ~45°. Figure 5c1 shows the distribution of α nanoprecipitates along the rolling direction in the unstretched alloy. The corresponding EBSD image reveals the layered structure decorated with α nanoprecipitates, see Fig. 5c2. The elongated grains have different orientations. In general, the α/β interfaces in HLS alloys serve as preferential sites for the initiation and propagation of microcracks owing to the strain incompatibility between α and β phases caused by their strength discrepancy (see Supplementary Fig. 17). At post-uniform elongation with ε = 0.26, indeed the microcracks initiated at the α/β interfaces and were arrested by the adjacent intragranular α_*Grain*_ nanoprecipitates, as marked white arrows in Fig. 5c3, and the crack tip became blunted. This crack-tip blunting effect via α_Grain_ particles was verified by the EBSD results (see the inset in Supplementary Fig. 18a, b). For the HLS-0.43 alloy at ε = 0.32, some arrested cracks were deflected (cyan arrows) and branched (green arrows) during their propagation, and even entered the adjacent β layer, see Fig. 5c4 and Supplementary Fig. 18c. The flat specimen is prone to additional localized necking after large strains^56^, leading to nonnegligible shear strains/stresses. The stress concentration at the crack tip arrested by α*-*nanoprecipitates cannot be released. This process does not facilitate increasing microcrack density (i.e., the number density of cracks per unit area), because α nanoprecipitates can trigger the TRIP mechanism to release local stress concentrations on the one hand, and shield the stress field of a crack tip, thus result in crack deflection on the other hand. Therefore, the crack branch intensively took place (Fig. 5c4), and these abundant secondary cracks also began to propagate along the α/β layer interfaces, i.e., multiple delamination, as shown in red arrows in Fig. 5c5 and Supplementary Fig. 18d. Fortunately, these cracks can be obstructed by the α phase (including α_Int_ and α_GB_) on the opposite interface/boundary to delayed fracture. In such a case, the microcrack density notably increased in this stage to dissipate the strain energy, see Supplementary Fig. 18. For this HLS-0.43 alloy at a strain (ε = 0.36) near the fracture strain, the microcrack extends intensively, as marked by the yellow dashed lines in Fig. 5c5, leading to massive α/β interfacial delamination. From our characterization of the locations at delamination crack tips, the α_Int_/β interfaces in our alloy serve as preferential sites for the initiation and propagation of delamination microcracks. Taken together, the fracture process is schematically shown in Fig. 5d. The delamination toughening mechanism associated with intensive but controlled cracking at α/β layer interfaces normally to the primary fracture surface dramatically enhances the overall fracture resistance to avoid catastrophic fracture. This unique fracture behavior of our HLS Ti alloys is similar to that observed in a medium Mn steel^24^ to some degree, which relies on both TRIP and delamination toughening to boost the fracture properties.

## Discussion

Compared with other heterostructures, our HLS alloys include the following unique features that are essential for creating the observed improved mechanical behavior^21^. (i) The submicron-lamellar microstructure, which enables the operation of ODP in lieu of SIM in the metastable matrix at low stresses/strains to realize high yield strength. (ii) The multi-morphological α-nanoprecipitates are efficient in constraining the plastic deformation of the matrix and triggering SIM via high-stress concentrations to maintain high strength and ductility. (iii) There are high densities of layer boundaries and hetero-phase β/α″ interfaces, where dislocations are stored to enhance the work-hardening capability of a material and thus the ductility.

We start with an explanation of the high strength and exceptional ductility of our designed HLS Ti alloys. First, the high (yield) strength, in addition to solid solution strengthening, is expected mainly from the multi-morphologic nanoprecipitates, preexisting dislocations, and ultrafine layers/grains. For instance, the strengthening contribution of α nanoprecipitates is approximately 60 MPa in the present HLS-0.43 β-Ti alloys. The strength contribution from preexisting dislocations, not only serving as sources for new dislocations^23,57^, but also acting as barriers for gliding dislocations as well as martensite interfaces to some degree^28^, is ~180 MPa in our HLS-0.43 alloys. More importantly, the contribution of submicron β layers/grains (interface/boundary strengthening) is ~365 MPa, which contributes most to the yield strength. For the detailed calculation process, please refer to the theoretical calculations of strength in Supplementary Note 9.

**Fig. 6:**
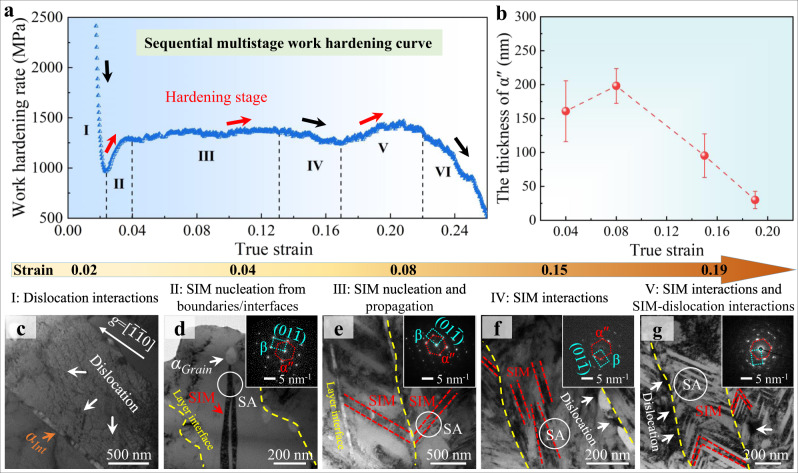
The multistage work-hardening behavior and the underlying mechanisms in HLS-0.43 β-Ti alloys. **a** A representative work-hardening rate curve of HLS-0.43 β-Ti alloys. **b** Profiles of martensitic thickness *vs*. true strain, revealing a maximum thickness of martensitic plates at a strain of ε = 0.08. Below this strain, martensites have just nucleated so that they can grow up to large thickness with increasing ε; while beyond this strain these submicron-sized martensites interplay, leading to microstructural refinement and the formation/nucleation of nanomartensites. **c**–**g** TEM images show the mechanisms of multistage work-hardening in HLS-0.43 β-Ti alloys: **c** dislocation-interface interactions, ε = 0.02; **d** SIM nucleation from the α/β interface/boundary, ε = 0.04; **e** SIM nucleation from the α/β interface and propagation, ε = 0.08; **f** massive SIM interactions leading to refinement of submicron-sized martensitic plates, ε = 0.15; **g** SIM interactions and nanomartensite-dislocation interactions, ε = 0.19. Error bars indicate standard deviations for three statistics. Beam parallel to a <011>_β_ zone axis in **d**–**g**.

Next, we explain in more detail the large uniform elongation of HLS β-Ti alloys (except the HLS-0.34 alloy). The high work-hardening rate *θ* accompanying the elevating stresses seen in the tensile curve (Supplementary Fig. 11) is essential for achieving large uniform elongation without pronounced strain localization such as necking. It is found that EGS and HLS-1.2 β-Ti alloys exhibit the typical three-stage *θ*, similar to most reported TRIP β-Ti alloys^20,52^. In contrast, HLS-0.43 β-Ti alloys show a unique multistage *θ*, similar to some reported heterostructured alloys^21,58,59^. Our HLS-0.34 β-Ti alloys show a monotonically reduced one-stage *θ* with a limited ε_f_ of ~3%. The discrepancy in the work-hardening rate among the present β-Ti alloys implies that the work-hardening mechanisms are not only strongly *h*_*β*_-dependent, but also evolve with plastic strains. In what follows, we take the HLS-0.43 β-Ti alloy as an example to uncover the underlying mechanisms for this unique multistage work-hardening rate for the uniform elongation ε_U_, as shown in Fig. 6.

In our HLS-0.43 β-Ti alloys, the soft-metastable β-layers first undergo deformation via ODP. The dislocation interactions and dislocation-interface interactions are thus attributed to the sharply reduced *θ* in stage-I, see Fig. 6a. This is because the strong size (submicron β layers/grains) constraining effect renders the stress is insufficient to trigger SIM^31,60^; instead ODP is first activated to accommodate plastic strains (see Fig. 6c). As the stress increases in stage-II, SIM begins to nucleate from α/β interfaces and grows up to a submicron-sized martensitic plate, see Fig. 6b, d and Fig. 1c1-c2. In other words, it is the ODP to SIM transition that results in the increased fraction of SIMs, which contributes to the enhanced *θ*. In stage III, with slightly increased *θ*, numerous SIMs intensively nucleate/propagate inclined to α/β interfaces and become thicker on the order of ~200 nm, leading to the refined microstructure, see Fig. 6b, e. Further increasing the stress in stage IV, pronounced martensite interactions result in a much-refined substructure with ~100-nm-thick martensites and nanosized β-blocks^28^, see Fig. 6b, f. Despite this heterogeneous nanostructure containing more interfaces/boundaries that are favorable for high *θ*, it, in turn, renders the suppression of martensites^28^ at this stress level, and dislocation takes place associated with reduced *θ*, see Fig. 6f. Therefore, these two competing factors render the reduction in *θ* in this stage. In stage V, more severe deformation not only refines nanomartensites into smaller ones with an average thickness of ~30 nm associated with notably increased β/α″ interfaces, but also promotes the formation of hierarchical α″ nanovariants (verified by the corresponding diffraction ring) in the zig-zag pattern, see Fig. 6g. Thus, the SIM-related defect interactions^28,61^ coupled with dislocation-interface interactions^21,28^ induce high *θ*. This hierarchically heterogeneous microstructure becomes the root of notably increased *θ*. Obviously, our design strategy of sequential activation of plastic carriers utilizing the multi-morphologic α nanoprecipitates is also very efficient in enhancing the uniform elongation of materials.

Finally, we elucidate the unique fracture behavior caused by our structural design, i.e., the post-uniform elongation stage of this HLS β-Ti alloy. In fact, the metastable matrix has desired resistance to crack growth, due to the activation of hierarchical SIM plates via local stress concentrations raised by α nanoprecipitates^62^ that can serve as crack buffers on the microcrack tips. The release of the local stress field of a crack tip via the BCC β-to-orthorhombic α″ structural transition effectively suppresses crack initiation and propagation^61^. The resultant SIM plates in a zig-zag pattern with a high density of martensite interfaces could suppress the propagation of microcracks as well. Specifically, these α nanoprecipitates located at different positions can hinder crack propagation, and cause crack deflection/branch, even trigger multi-delamination behavior at very large strains/stresses. In this regard, fracture under the plane-strain condition is automatically transformed into a series of fracture processes of individual β-layers in the plane-stress condition through the thickness. Therefore, compared with EGS samples, no crack percolation or catastrophic failure event occurred in our transformable HLS alloys (with *h*_*β*_ > 0.34 μm)^63^, irrespective of their high densities of microcracks. In this work, our structural design combining lamination decorated with hard-yet-deformable nanoprecipitates and metastability offers an available pathway to develop strong alloys with enhanced ductility and superior fracture resistance.

In summary, we proposed an innovative design strategy by engineering hierarchal multifunctional nanoprecipitates in heterogeneous metastable alloy systems to achieve desired strength-ductility synergy for superior fracture resistance at ambient temperature. It is demonstrated that tuning the characteristics (such as size, spacing, morphology) of trifunctional nanoprecipitates without change of the alloy composition to control the activation sequence of deformation mechanisms (e.g., SIM and ODP) can substantially ductilize and toughen metastable materials with enhanced (yield) strength. This alloy design strategy can also be feasibly applied to many other second-phase-reinforced metastable alloy systems (e.g., conventional TRIP steels and multicomponent alloys) in a transformable matrix to achieve desired properties for specific applications.

## Methods

### Sample preparation

The Ti-1Al-8.5Mo-2.8Cr-2.7Zr (wt%) alloy was prepared from pure elements by arc melting, and was remelted at least five times to guarantee chemical homogeneity in a high-purity argon atmosphere. The ingot was cast into a 14 × 10 × 55 mm^3^ water-cooled copper mold. Homogenization was performed at 1273 K for 60 min in an Ar atmosphere followed by water quenching, and the β-transus temperature (${T}_{\beta }$) of the present Ti-Al-Mo-Cr-Zr alloy was measured to be ~1040 ± 5 K by metallographic analyses. The chemical composition was determined to be 8.57 Mo, 2.69 Cr, 2.83 Zr, 1.1 Al, 0.004 C, 0.0041 H, 0.11 O, 0.007 N, and the balance Ti (wt%), using inductively coupled plasma (ICP) mass spectrometry. The homogenized specimen was then subjected to beta transus rolling. The height reduction for each pass was controlled to be ~0.3 mm, and the accumulative rolling reduction ranged from 78 to 93%. After rolling, the specimens were solution treated at 1050 K for three different durations, e.g., 1, 30, and 60 min. Based on various combinations of total rolling reduction and duration of the solution treatment, specimens with six distinct HLS structures (named HLS-0.34, HLS-0.43, HLS-0.69, HLS-1.2, HLS-2.7, and HLS-3.2 hereafter, according to the corresponding β-layer thickness in the specimen, see Supplementary Fig. 5) and two EGS (named EGS-24 and EGS-61 hereafter, according to the corresponding β grain size in the specimen, see Supplementary Fig. 19) structures were prepared.

#### Mechanical properties test

Dog-bone specimens with gauge dimensions of 15 × 3.2 × 0.5 mm^3^ were cut parallel to the rolling direction (RD) for quasi-static uniaxial tensile tests. The experiment was performed on an Instron 5969 universal testing machine at room temperature, with an initial strain rate of 1 × 10^−3^ s^−1^. All tests were repeated at least five times to ensure data reproducibility. The hardness of the β matrix and α/β regions was measured using a TI950 TriboIndenter (Hysitron, Minneapolis, MN) with a standard Berkovich tip at room temperature, following the Oliver-Pharr method. The hardness test was conducted in load-controlled mode with a prescribed loading of ~3000 μN under a loading time of 5 s, corresponding to the loading strain rates of ~0.1 s^−1^. The holding time is 2 s, and the unloading time was 5 s. To improve the reliability and accuracy of the present measurements, great efforts were devoted to the correction of thermal drift in the nanoindentation test. In the present study, the allowable drift rate was set at 0.02 nm s^−1^, which is fivefold smaller than the typical value (0.1 nm s^−1^) generally used in typical nanoindentation compression tests.

#### Microstructural characterization

Prior to mechanical testing, all samples were mechanically ground at least 0.2 mm, followed by polishing and etching so as to inspect deformation surface morphology and eliminate the possible influence of the oxidation effects during hot rolling. A scanning electron microscope (SEM, JSM-7001F) was used to characterize the microstructure of the HLS before and after the tensile tests. The lengths along the major and minor axes of different types of α particles (e.g., the α particles in interfacial layers (α_Int_), the nearly equiaxed intragranular α (α_Grain_) particles, and the intergranular α (α_GB_) particles) were measured separately by linear intercept method using image-pro software. The statistical distribution was obtained based on the measurement of at least 1000 particles. The heterogeneous laminated structures were also characterized by electron back-scattered diffraction (EBSD). EBSD measurements were carried out in a field emission SEM (JSM-7001F) equipped with an automatic orientation acquisition system, and the EBSD data was post-processed using Channel 5. A transmission electron microscope (TEM, JEM-2100) operated at 200 kV was employed to reveal the microstructural features.

## Supplementary information

Supplementary Information

## Data Availability

The data that support the findings of this study are available from the corresponding author upon request.
