# Supplementary material for: Trifunctional nanoprecipitates ductilize and toughen a strong laminated metastable titanium alloy
Source: Nat Commun. 2023 Mar 13;14:1397. doi: 10.1038/s41467-023-37155-y (PMC10011607; doi:10.1038/s41467-023-37155-y)
Supplement: Supplementary file 1 — Supplementary Information [file 41467_2023_37155_MOESM1_ESM.pdf]

Supplementary information for

Trifunctional nanoprecipitates ductilize and toughen a strong laminated metastable titanium alloy

Chongle Zhang§, Shuaiyang Liu§, Jinyu Zhang*, Dongdong Zhang, Jie Kuang, Xiangyun Bao, Gang Liu, Jun Sun*

State Key Laboratory for Mechanical Behavior of Materials, Xi'an Jiaotong University, Xi'an 710049, People's Republic of China

§. These authors contribute equally to this work.

*E-mail address: jinyuzhang1002@mail.xjtu.edu.cn (Jinyu Zhang)

junsun@mail.xjtu.edu.cn (Jun Sun)

Contents:

Supplementary Figure 1. The composition design and microstructure of our designed Ti alloys
Supplementary Figure 2. Microstructural characterization of the designed Ti-1Al-*x*Mo-2.8Cr-2.7Zr (*x* = 6.5, 8.5 and 10.5) alloys after water quenched
Supplementary Figure 3. Mechanical responses and deformation mechanisms of our designed Ti alloys with different Mo contents.
Supplementary Figure 4. Thermomechanical processing scheme of the present HLS and EGS β-Ti alloys
Supplementary Figure 5. The distribution of the β layer-thickness in different HLS β-Ti alloys
Supplementary Figure 6. Microstructural features of the present HLS β-Ti alloys
Supplementary Figure 7. EBSD analysis of the present HLS β-Ti alloys
Supplementary Figure 8. The microstructure and the distribution of alloying elements in the present HLS-0.43 alloys
Supplementary Figure 9. Histograms showing the statistical information of the size and morphology of the β matrix, and the α phase in HLS-0.43 β-Ti alloys
Supplementary Figure 10. Bar charts showing the statistical distribution of the local misorientation in EGS-61 alloy.
Supplementary Figure 11. True stress-strain curves and the corresponding work hardening rate of the present HLS and EGS β-Ti alloys
Supplementary Figure 12. The yield strength vs. inverse β-layer thickness
Supplementary Figure 13. The plastic work density of the present β-Ti alloys
Supplementary Figure 14. Fracture behavior and the underlying toughening mechanisms of EGS without α nanoprecipitates β-Ti alloys.
Supplementary Figure 15. Fracture behavior and the underlying toughening mechanism of EGS with α nanoprecipitates β-Ti alloys.
Supplementary Figure 16. Mechanical responses of EGS samples without and with α nanoprecipitates.
Supplementary Figure 17. Nanoindentation hardness measurements for the HLS-0.43 β-Ti alloys
Supplementary Figure 18. Evolution of the microcrack density during tension in HLS-0.43 β-Ti alloys
Supplementary Figure 19. Mechanical responses of the present EGS β-Ti alloys and a comparison of the measured and calculated yield strength
Supplementary Figure 20. The definition of the spacing of intragranular and intragranular particles in HLS β-Ti alloys
Supplementary Table 1. Detailed rolling and heat treatment process for present HLS and EGS β-Ti alloys
Supplementary Table 2. Grains and α precipitates: statistical results
Supplementary Table 3. The dislocation densities of different HLS β-Ti alloys
Supplementary Table 4. Summary of the mechanical properties of the present β-Ti alloys

Supplementary Table 5. Summary of the mechanical properties of the reported β-Ti alloys
Supplementary Note 1. Composition design
Supplementary Note 2. Thermomechanical processing to prepare HLS alloys
Supplementary Note 3. Detailed microstructural analysis for the present HLS and EGS β-Ti alloys
Supplementary Note 4. The uniform elongation and yield strength vs. inverse β-layer thickness of the present β-Ti alloys
Supplementary Note 5. Plastic work density of the present β-Ti alloys
Supplementary Note 6. Fracture behavior and the underlying mechanism of EGS samples without and with α nanoprecipitates.
Supplementary Note 7. Nanoindentation testing for the strength discrepancy between α and β phases
Supplementary Note 8. Evolution of the microcrack density in HLS-0.43 β-Ti alloys
Supplementary Note 9. Theoretical calculations of strengthening responses
Supplementary Note 10. Summary of the mechanical properties of the present and other β-Ti alloys
Supplementary References

Supplementary Note 1. Composition design

Based on the "*d*-electron design method" proposed by Morinaga and colleagues[1], the M_d = room temperature (RT) curve can be drawn on the $\overline{Bo}$-$\overline{Md}$ diagram[2,3], showing the position of alloys with the ability to exhibit the TRIP and TWIP effects simultaneously (the black dash line in Supplementary Fig. 1), such as Ti-12Mo[4], Ti-10Mo-5Nb[5], Ti-12Mo-3Zr[6], Ti-8.5Cr-1.5Sn[3], Ti-3Al-5Mo-7V-3Cr[7] and Ti-12V-2Fe-1Al[8] alloys. Therefore, the region around the M_d = RT curve is supposed to be a desired location for TRIP/TWIP β-Ti alloys. The TRIP of these alloys may have low critical stresses, i.e., low yield strength. Therefore, in order to increase the yield strength and activate TRIP at high stress levels to maintain high ductility, the alloy composition can be adjusted from M_d = RT towards the Slip/Twin curve in the $\overline{Bo}$-$\overline{Md}$ diagram, which can be expected by increasing the chemical stability of a TRIP/TWIP alloy, such as Ti-13Mo[9], Ti-10V-4Cr-1Al[10] and Ti-14V-2Fe-1Al[8] alloys.

On the other hand, there naturally exists a boundary between TRIP/TWIP and Slip in the $\overline{Bo}$ - $\overline{Md}$ map, across which the deformation mechanism will be transformed from one to the other. Most of TRIP/TWIP alloys are located between M_d = RT and Slip/Twin lines, consistent with the empirical border in Supplementary Fig. 1. Nevertheless, it is interesting to witness that some alloys still activate the TRIP mechanism even though they are located at the Slip/Twin boundary[9]. For example, the Ti-13Mo alloy located at the Slip/Twin boundary still activates the stress-induced martensite (SIM), while the deformation mechanism of both Ti-14Mo and Ti-15Mo alloys is twinning (at small strains), see Supplementary Fig. 1. In other words,

although these alloys likely have the highest chemical stability located at the Slip/Twin boundary, the TRIP operates at high stresses. Thus, we attempted to design a transformable alloy with the composition at the Slip/Twin boundary.

Here, based on previous (TRIP/TWIP) and (TWIP/Slip) Ti alloys (see Supplementary Fig. 1), we designed the present novel β-Ti alloys. In our alloys, the addition of alloying elements Mo, Cr, Al and Zr are mainly based on the following three reasons. (i) The first one is the solid solution strengthening. The β-stabilizing isomorphous elements Mo and eutectoid elements Cr can remarkably strength β matrix[7] and refine the grain sizes[11,12]. Moreover, Al as the α-stabilizer can also strengthen the alloy, especially the α nanoprecipitates (Al is α stability element). The hard α nanoprecipitates are expected to further increase the local stress levels during deformation to activate TRIP[13]. Zr has been regarded as a neutral element in Ti alloys, and it is infinitely solid solution in both α-Ti and β-Ti alloys[11,14], which can improve strength without reducing plasticity[14]. (ii) The second is to make sure that the $\overline{\mathrm{Bo}}$ and $\overline{\mathrm{Mo}}$ values are located at the Slip/Twin boundary. This allows the alloy with the highest β stability to activate SIM and thus obtain high strength. (iii) The third is to adjust the position of the β matrix in the $\overline{\mathrm{Bo}}$-$\overline{\mathrm{Md}}$ diagram based on Ti-Mo and Ti-Cr alloy systems. As shown in Supplementary Fig. 1, the β-stabilizing isomorphous elements Mo and the eutectoid element Cr result in the alloying vectors up-left and down-left, respectively. To ensure the SIM rather than the stress-induced twinning or quenched martensites can be obtained, we fabricated three Ti-1Al-*x*Mo-2.8Cr-2.7Zr (wt.%, x = 6.5, 8.5 and 10.5) alloys with different Mo contents, as shown in

Supplementary Fig. 1.

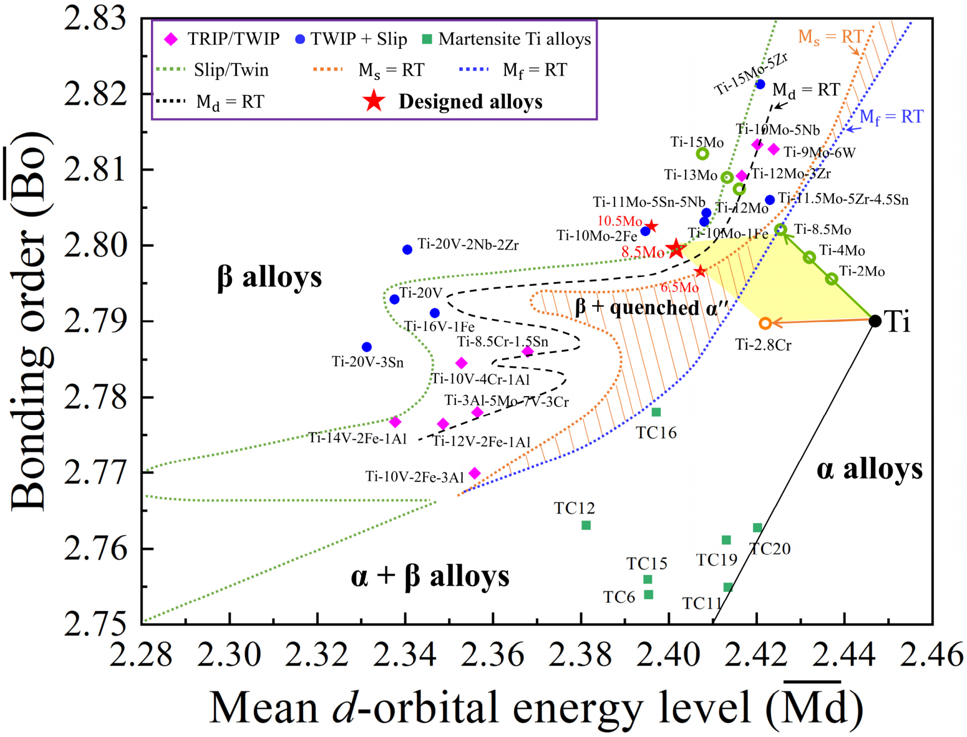

Supplementary Figure 1 The composition design and microstructure of our designed Ti alloys. The composition based on the *d*-electron design map[1,15,16] shows the position of Ti-1Al-*x*Mo-2.8Cr-2.7Zr (*x* = 6.5, 8.5 and 10.5) alloys.

To obtain similar microstructures, all these designed alloys were processed and heat-treated in the same way based on the β-transus temperature. For example, these Ti-1Al-*x*Mo-2.8Cr-2.7Zr (*x* = 6.5, 8.5 and 10.5) alloys underwent the 60 min solution treatment (all are 5~10 K higher than the β-transus temperature) showed the equiaxed-grain structured (EGS) feature**.** Supplementary Fig. 2 shows the initial microstructures of the present Ti-1Al-*x*Mo-2.8Cr-2.7Zr (*x* = 6.5, 8.5 and 10.5) alloys, revealing the Ti-1Al-8.5Mo-2.8Cr-2.7Zr and Ti-1Al-10.5Mo-2.8Cr-2.7Zr alloys have equiaxed β grains, while the Ti-1Al-6.5Mo-2.8Cr-2.7Zr alloy is composed of β and quenched martensite phases. Supplementary Fig. 3a shows the tensile engineering

stress-strain curves of Ti-1Al-*x*Mo-2.8Cr-2.7Zr alloys at the quasi-static strain rate. Although Mo doping can induce solid solution strengthening, the yield strength of 6.5Mo alloys is much more than that of 8.5Mo alloys. Obviously, the contribution of yield strength mainly origins from the martensite phase boundaries strengthening. Compared with 10.5Mo sample, 8.5Mo sample exhibited a relatively lower yield stress but followed by superior ductility, similar to previous TRIP/TWIP β-Ti alloys[3-5,17]. Further, Supplementary Fig. 3b-c shows the typical surface morphologies of the stretched 8.5Mo alloy. The plate-like deformation bands were identified to be stress-induced martensite (SIM) α″, as verified by the corresponding α″ phase map, implying the SIM is the initial deformation mechanism during deformation. Often, the critical twinning stress increases with increasing the Mo content-dependent β-stability, thus the yield strength of 10.5Mo alloys is enhanced, while their ductility is significantly decreased. On the other hand, 10.5Mo alloy is located on the left of Slip/Twin boundary (i.e., in the Slip region), similar to Ti-10Mo-2Fe[18], Ti-20V-3Sn[19] and Ti-20V-2Nb-2Zr[20] alloys, see Supplementary Fig. 1. Subsequently, the activated deformation productions are identified to be $\{332\}_\beta\langle 113\rangle_\beta$ twins (Supplementary Fig. 3d-e) with a characteristic misorientation of 50.5° around $\langle 110\rangle_\beta$ axis[7,21]. This is evidenced by the misorientation angle along the 'AB' line crossing a twin in Supplementary Fig. 3d. Therefore, we only focus on the microstructure and mechanical properties of Ti-1Al-8.5Mo-2.8Cr-2.7Zr alloys to convey our TRIP/TWIP alloy design strategy, which provides guidance to design of other alloys with the same nature of our alloys.

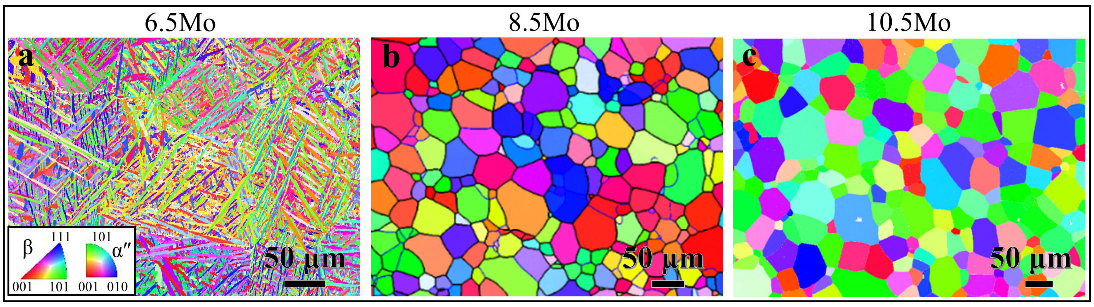

Supplementary Figure 2 Microstructural characterization of the Ti-1Al-*x*Mo-2.8Cr-2.7Zr (*x* = 6.5, 8.5 and 10.5) alloys after water quenching. **a** The EBSD image shows quenched martensites in the Ti-1Al-6.5Mo-2.8Cr-2.7Zr alloy. **b** and **c** EBSD images show the equiaxed β grain structure in Ti-1Al-8.5Mo-2.8Cr-2.7Zr and Ti-1Al-10.5Mo-2.8Cr-2.7Zr alloys, respectively.

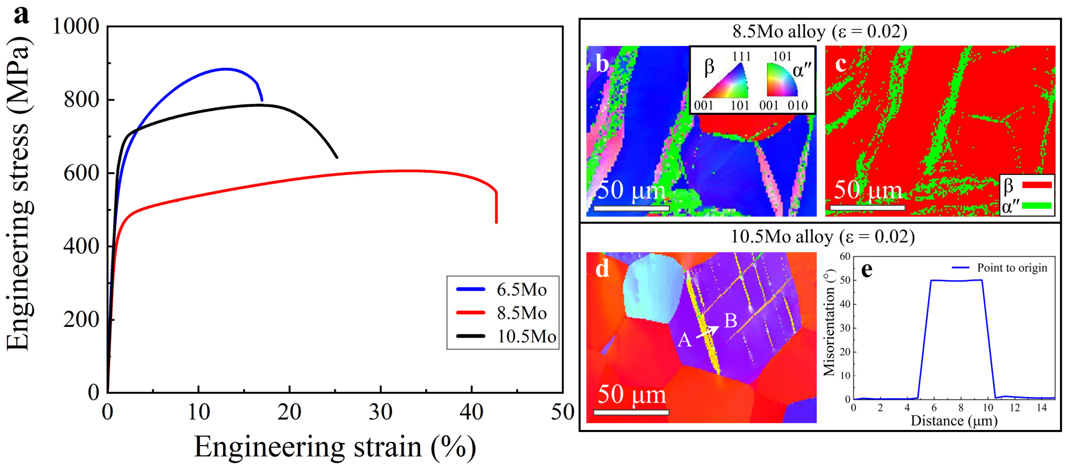

Supplementary Figure 3 Mechanical responses of our designed Ti alloys with different Mo contents. **a** Tensile engineering stress-strain curves of Ti-1Al-*x*Mo-2.8Cr-2.7Zr (*x* = 6.5, 8.5 and 10.5) alloys at room temperature. **b**, **c** Surface morphologies of Ti-1Al-8.5Mo-2.8Cr-2.7Zr stretched samples with the strain of 0.02. **c** The Inverse pole figure (IPF) image shows the plate-like deformation bands that were identified to be SIM α″, as verified by the corresponding α″ phase map. **d** Surface morphologies of Ti-1Al-10.5Mo-2.8Cr-2.7Zr stretched samples with the strain of 0.02. The IPF image shows $\{332\}_{\beta}\langle 113\rangle_{\beta}$ twins. **e** Misorientation profile along the AB line crossing a twin in **d**.

Supplementary Note 2. Thermomechanical processing to prepare HLS and EGS β-Ti alloys

In this study, we proposed cyclic hot-rolling & short-time-solutioning (HR&SS) processing to fabricate a duplex heterogeneous submicron-laminated structure (HLS) metastable β-Ti alloy, as shown in Supplementary Fig. 4. The samples were first rolled above the β-transus temperature (T_{β} ~1040 ± 5 K). Dynamic recrystallization of β grains takes place during thermomechanical processing so that the β-grains are notably refined. These refined β-grains are maintained due to the constraining effect of the α phase, which is precipitated from the β-matrix when the rolling temperature is reduced to the (α+β) two-phase field. After each rolling pass with a reduction in thickness of 3%, the specimen was heated to the furnace temperature (~1050 K) for ~1 min. The HR&SS processes were repeated until the total thickness reduction of the specimen reached 78 - 93%, see Supplementary Table 1. Finally, the HR&SS sample was performed at 1050 K for 1 min, followed by water quenching (WQ). Obviously, HR&SS processing was adopted to retain a low fraction of α nanoprecipitates without remarkably altering the phase stability of the β-matrix during subsequent WQ and to have almost the similar dislocation density in all HLS samples with different β layer thickness (h_{β}). A long-time solution treatment (for ~30 to 60 min) was employed to prepare EGS alloys with low dislocation densities (see Supplementary Table 3).

It should be emphasized that the β layer thickness is controlled by the rolling temperature, holding time and rolling reduction. Therefore, it is critical to control the rolling or forging reduction to realize the desired layer thickness in industry by decreasing the total thickness of industrial samples to about several to tens of

millimeters. Since the thermo-mechanical processing to architect the HLS structure based on the β-transus rolling is industrially accessible, therefore this process can be employed to prepare industrial scale Ti alloys.

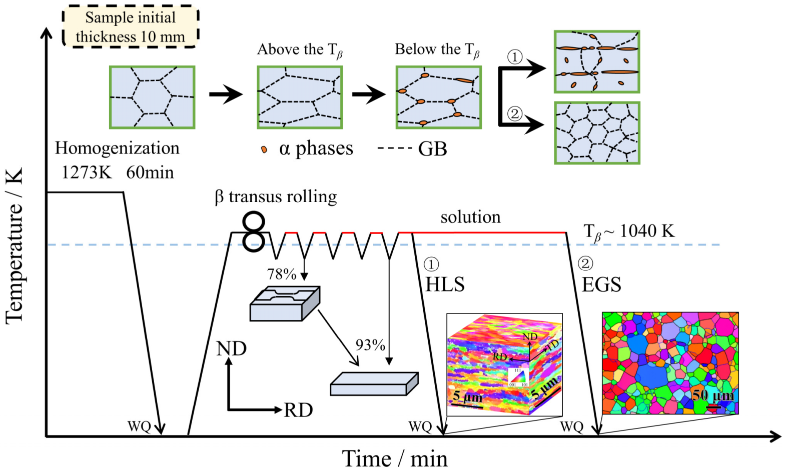

Supplementary Figure 4 Thermomechanical processing scheme of the present HLS and EGS β-Ti alloys, together with the schematic illustration showing the typical microstructural evolution. By combining the total rolling reduction and duration of the solution treatment, duplex heterogeneous submicron-laminated structured (HLS) alloys with different thicknesses h_β were prepared. The insets show the 3D stereographic microstructure of the HLS-0.43 alloy reconstructed by electron backscatter diffraction (EBSD) IFP maps and the EBSD image of the equiaxed-grain structured (EGS) sample.

Supplementary Table 1 Detailed rolling and heat treatment process for present HLS and EGS β-Ti alloys.

Sample	Reduction in thickness	Cycle number	Solution time
EGS-61	93%	31	60 min
EGS-24	93%	31	30 min
HLS-3.2	78%	26	1 min
HLS-2.7	81%	27	1 min
HLS-1.2	84%	28	1 min
HLS-0.69	87%	29	1 min
HLS-0.43	90%	30	1 min
HLS-0.34	93%	31	1 min

Supplementary Note 3. Detailed microstructural analysis for the present HLS and EGS β-Ti alloys

The present HLS β-Ti alloys possess the key combination of three characteristics, i.e., lamination, multiple phases and metastability. More details about the microstructural features, including the layered structure with different thicknesses h_β, crystallographic orientations, sizes of α precipitates located at different sites, density of geometrically necessary dislocations (GNDs), and distribution of alloying elements are displayed in Supplementary Figs. 5-9 and Supplementary Tables 2-3.

Supplementary Fig. 5 shows the layer thickness h_β distribution of the present HLS β-Ti alloys. The average layer thickness can be obtained from the Gaussian function by fitting these data. Supplementary Fig. 6 presents the microstructural features of the HLS β-Ti samples with different h_β. In the layer thickness direction, there is almost only one submicron-sized elongated β-grain along the rolling direction, i.e., the β-layer is almost composed of single layered grains, associated with some residual dislocations. The thickness (h_α) of α_{Int} is almost the same in all HLS β-Ti alloys, while the volume fraction (f), the average size (d) and the average spacing (λ) of both α_{Grain} and α_{GB} precipitates change with h_β, see Supplementary Table 2.

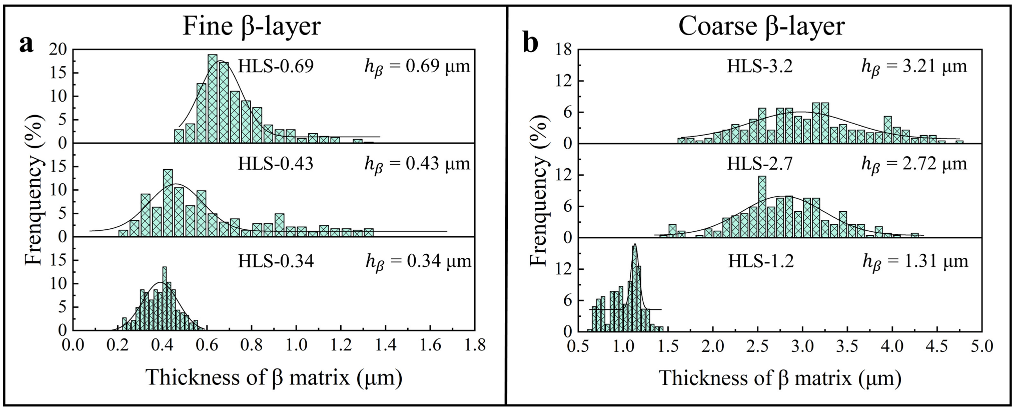

Supplementary Figure 5 Distribution of β layer-thickness (h_β) in all HLS alloys. **a**

The HLS structure with fine β-layers i.e., HLS-0.34, HLS-0.43, and HLS-0.69; **b** The HLS structure with coarse β-layers. i.e., HLS-1.2, HLS-2.7, and HLS-3.2.

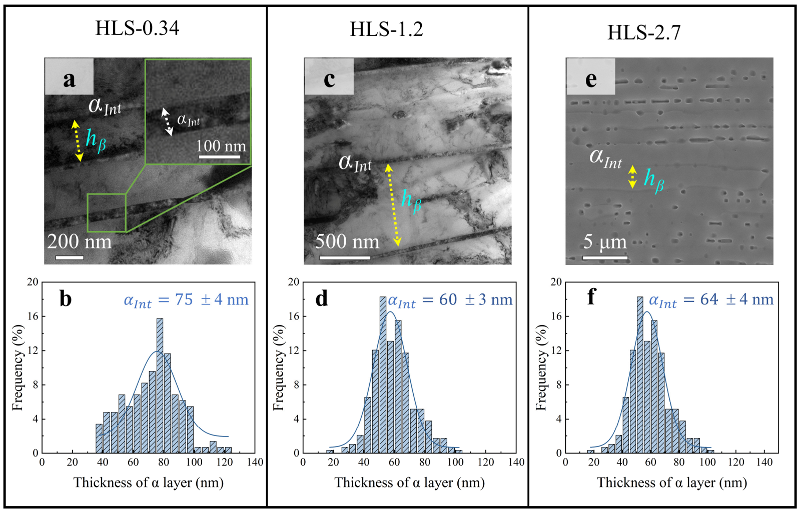

Supplementary Figure 6 Microstructural features of the present HLS β-Ti alloys. **a**, **c** and **e** Representative TEM/SEM images showing the layer structure in three typical HLS β-Ti alloys. **b**, **d** and **f** The statistical distribution of the interfacial α (α_{Int}) layer thickness. **a** and **b** correspond to HLS-0.34; **c** and **d** HLS-1.2; **e** and **f** HLS-2.7. The β layer-thickness (h_β) and the interfacial α (α_{Int}) layer-thickness are marked by yellow and white dotted arrows, respectively.

Supplementary Table 2 Grains and α precipitates: statistical results

Parameters (Symbol/Unit)	HLS-0.34	HLS-0.43	HLS-0.69	HLS-1.2	HLS-2.7	HLS-3.2
Average cross-sectional grain size (*d* / nm)	342 ± 19	431 ± 17	698 ± 23	1310 ± 45	2720 ± 31	3210 ± 68
Average aspect ratio of grain size	3.2 ± 0.5	2.3 ± 0.4	2.8 ± 0.3	2.4 ± 0.3	2.5 ± 0.2	2.1 ± 0.3
Average intragranular α_{Grain} diameter (nm)	151 ± 7	156 ± 6	178 ± 10	192 ± 13	185 ± 8	174 ± 6
Average aspect ratio of intragranular α_{Grain}	1.3 ± 0.2	1.3 ± 0.1	1.2 ± 0.2	1.4 ± 0.3	1.2 ± 0.1	1.5 ± 0.3
Volume fraction of intragranular α_{Grain} ($f\alpha_{Grain}$ / %)	1.2 ± 0.2	2.3 ± 0.4	2.5 ± 0.3	3.1 ± 0.2	3.5 ± 0.2	2.7 ± 0.3
Average intergranular α_{GB} diameter (nm)	185 ± 8	173 ± 12	195 ± 11	200 ± 9	210 ± 10	198 ± 13
Average aspect ratio of α_{GB}	1.1 ± 0.1	1.2 ± 0.1	1.4 ± 0.3	1.3 ± 0.2	1.4 ± 0.2	1.3 ± 0.3
Average intragranular α_{GB} spacing ($\lambda\alpha_{GB}$ / nm)			~ the β layer thickness			
Volume fraction of intragranular α_{GB} ($f\alpha_{GB}$ / %)	1.8 ± 0.2	2.2 ± 0.3	2.3 ± 0.2	2.7 ± 0.4	2.8 ± 0.3	3.1 ± 0.4
Average thickness of interfacial α_{Int} (nm)	75 ± 4	85 ± 5	89 ± 4	60 ± 3	64 ± 4	78 ± 6
Average length of interfacial α_{Int} (nm)	249 ± 11	210 ± 15	209 ± 9	195 ± 13	174 ± 8	159 ± 12
Average aspect ratio of interfacial α_{Int}	3.6 ± 0.4	3.1 ± 0.3	288 ± 0.2	2.5 ± 0.2	2.4 ± 0.4	2.2 ± 0.2
Volume fraction of interfacial α_{Int} ($f\alpha_{Int}$ / %)	4.9 ± 0.4	3.8 ± 0.2	3.3 ± 0.3	2 ± 0.2	1.2 ± 0.4	1.5 ± 0.3

Supplementary Fig. 7 shows the EBSD analysis for our HLS alloys, revealing the layered structure with randomly oriented grains. All three HLS alloys show elongated β-grains aligned roughly parallel to RD, regardless of the layer thickness. The local misorientation, caused by the local lattice curvature, originates from the accumulation of abundant dislocations. Hence, the peak center shifts in the bar chart toward the high angle with decreasing layer thickness, indicating a negative correlation between the layer thickness and the dislocation density.

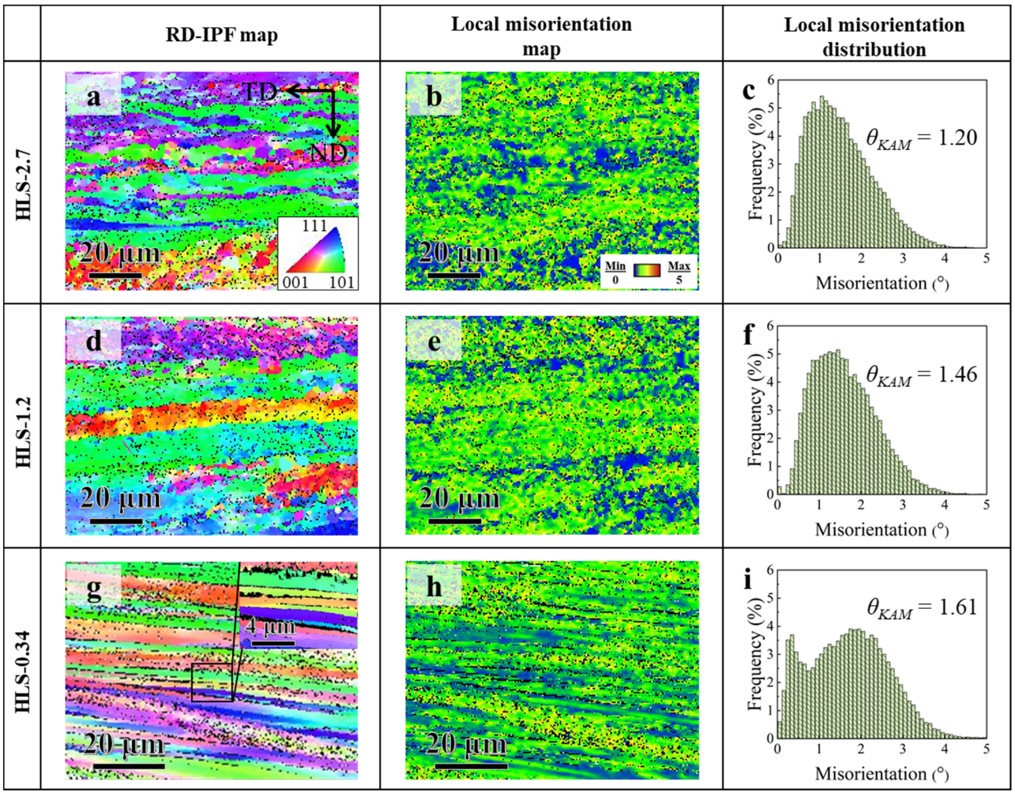

Supplementary Figure 7 EBSD analysis of the present HLS β-Ti alloys. **a**, **d** and **g** The RD-IPF maps; **b**, **e** and **h** The local misorientation maps (i.e., the kernel average misorientation (KAM) maps); The density of geometrically necessary dislocations (GNDs) obtained from the average KAM analysis for the HLS β-Ti alloys, see **Table S2**. **c**, **f** and **i** Bar charts showing the statistical distribution of the local misorientation. **a**, **b** and **c** correspond to HLS-2.7 alloys; **d**, **e** and **f** correspond to HLS-1.2 alloys; **g**, **h** and **i** correspond to HLS-0.34 alloys.

The dislocation (i.e., GND) density ρ was calculated based on the EBSD results using the following formula[22,23]:

$$\rho = \frac{2\theta_{KAM}}{Xb} \qquad (1)$$

where X is the step size. θ_{KAM} is the average KAM value of the specimen, given by:

$$\theta_{KAM} = \Sigma f_i \, \theta_i \qquad \theta<5° \qquad (2)$$

where θ_i and f_i are the local misorientation and its frequency, respectively. It is found that there is an increasing trend of the dislocation density with decreasing h_β, as listed in Supplementary Table 3.

Supplementary Table 3 The θ_{KAM} values and dislocation densities of different HLS β-Ti alloys.

Material	θ_{KAM} (°)	ρ (m^{-2})	σ_ρ (MPa)
HLS-3.2	1.13	2.82×10^{14}	153.9
HLS-2.7	1.20	3.01×10^{14}	158.6
HLS-1.2	1.46	3.64×10^{14}	174.9
HLS-0.69	1.53	3.81×10^{14}	179.1
HLS-0.43	1.56	3.89×10^{14}	180.9
HLS-0.34	1.61	4.00×10^{14}	183.7

Taking the HLS-0.43 Ti alloy as an example, there is a clear modulated structure composed of submicron-sized β layers and α_{Int} layers with distinguished α/β interfaces, see Supplementary Fig. 8. It is found that the β-stabilizing elements Cr and Mo are enriched in the β matrix, while the α-stabilizing elements Al is slightly enriched in α precipitates, see the EDS line scanning in Supplementary Fig. 8. The neutral element Zr is homogeneously distributed. The corresponding EDS analysis for a typical region containing α/β interfaces and different morphologies of α precipitates, showing the distribution of Ti, Al, Mo, Cr, and Zr from the black rectangle region in Supplementary Fig. 8c, is consistent with the line scanning result. The volume

fractions of α$_{Grain}$, α$_{GB}$ and α$_{Int}$ are ~2.3%, ~2.2% and ~3.8%, respectively. The average sizes of α$_{Grain}$ and α$_{GB}$ are 156 nm and 173 nm, respectively, as shown in Supplementary Fig. 9.

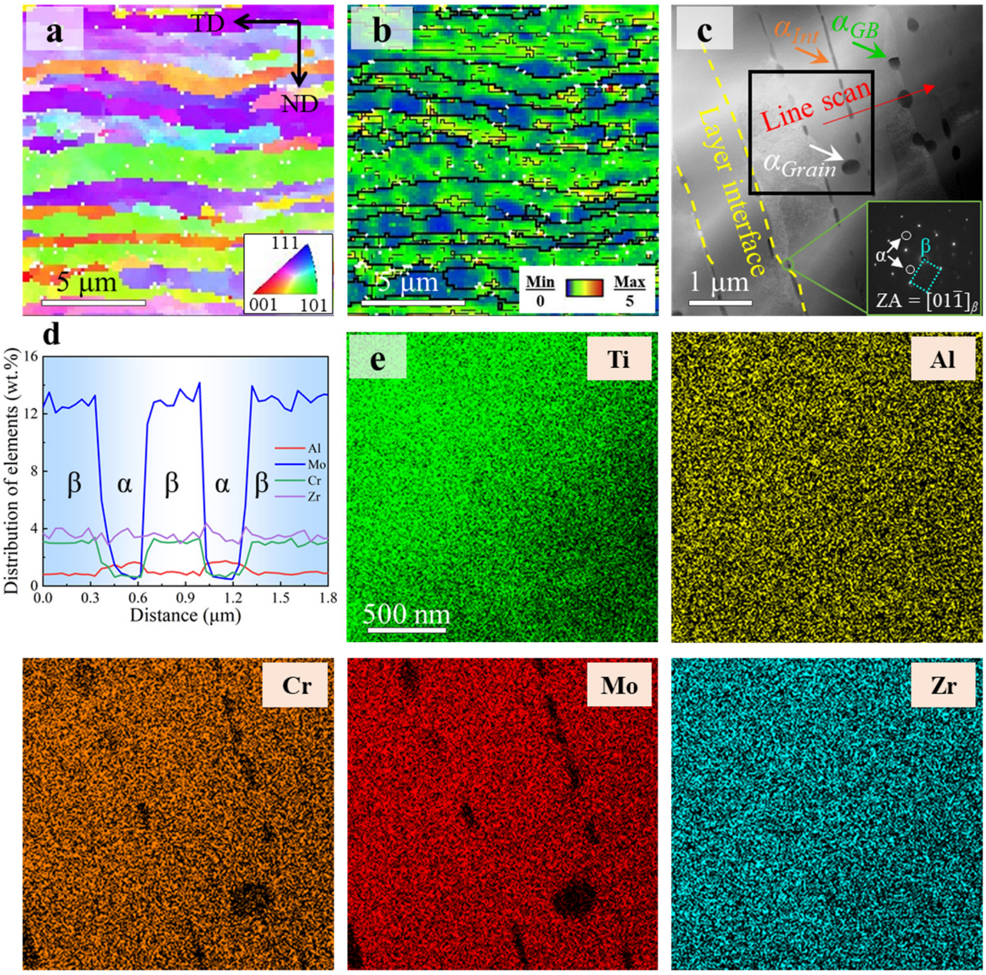

Supplementary Figure 8 The microstructure and the distribution of alloying elements in the present HLS-0.43 alloys. **a** The IPF image and **b** the corresponding KAM map for HLS-0.43 β-Ti alloys. TD, transverse direction; ND, normal direction. **c** A high-angle annular dark-field scanning TEM (HAADF-STEM) image of the HLS-0.43 alloy, showing multi-scaled α precipitates located at different positions, i.e., α_{Int}, α_{Grain} and α_{GB}, inidicated by arrows. The inset is the corresponding selected area electron (SAED) pattern. **d** EDS line scanning from the red arrow in **c**. **e** The corresponding EDS analysis for the black rectangular region in **c**.

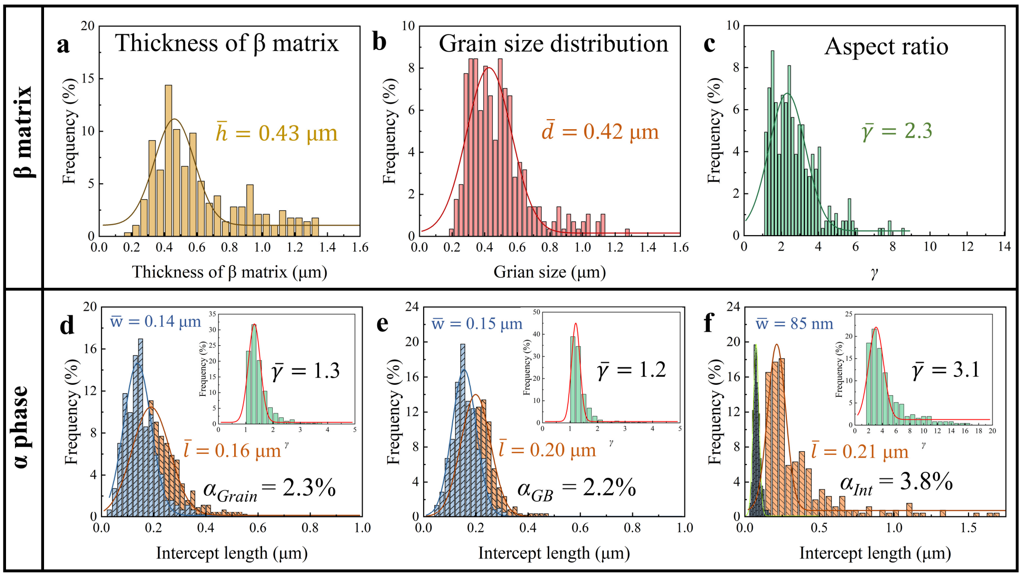

Supplementary Figure 9 Histograms showing the statistical information of the size and morphology of (**a-c**) the β matrix, and (**d-f**) the α phase in HLS-0.43 β-Ti alloys. **a** The β-layer thickness; **b** The β grain size; **c** The aspect ratio of β-grains; **d**, **e** and **f** The dimensions of intragranular α_{Grain}, intergranular α_{GB}, and interfacial α_{Int}, respectively. $\bar{l}$ and $\bar{w}$ represent the intercept length in the long axis and the short axis of the particle, respectively. The aspect ratio (γ) is defined as $\bar{l}/\bar{w}$.

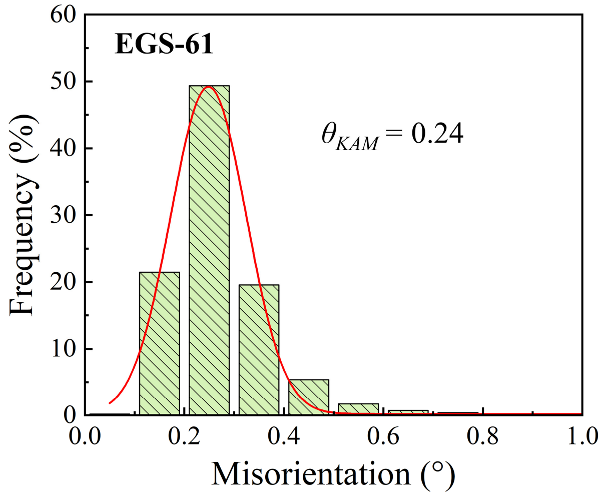

Supplementary Figure 10 Bar charts showing the statistical distribution of the local misorientation for EGS-61 alloy.

Supplementary Note 4. The uniform elongation and yield strength vs. inverse β-layer thickness of the present β-Ti alloys

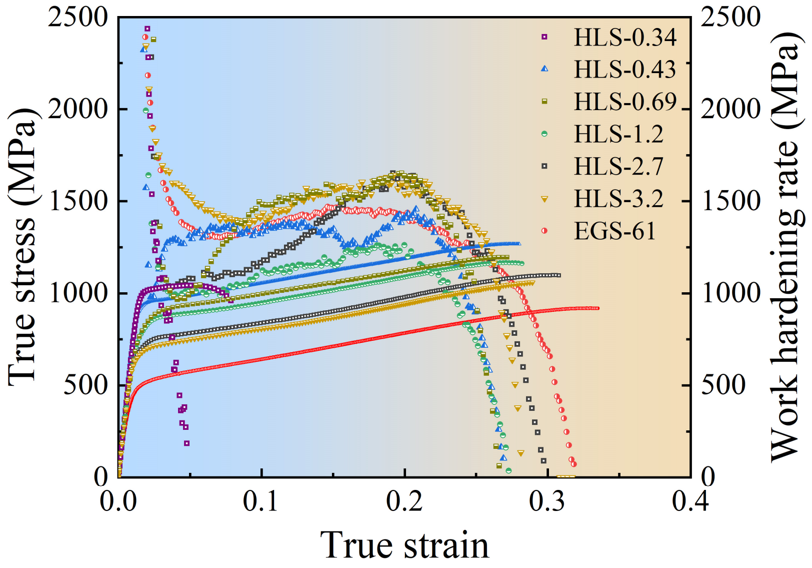

Supplementary Figure 11 True stress-strain curves and the corresponding work hardening rate of the present HLS and EGS-61 β-Ti alloys. The uniform elongation ε_U is determined based on Considère's criterion.

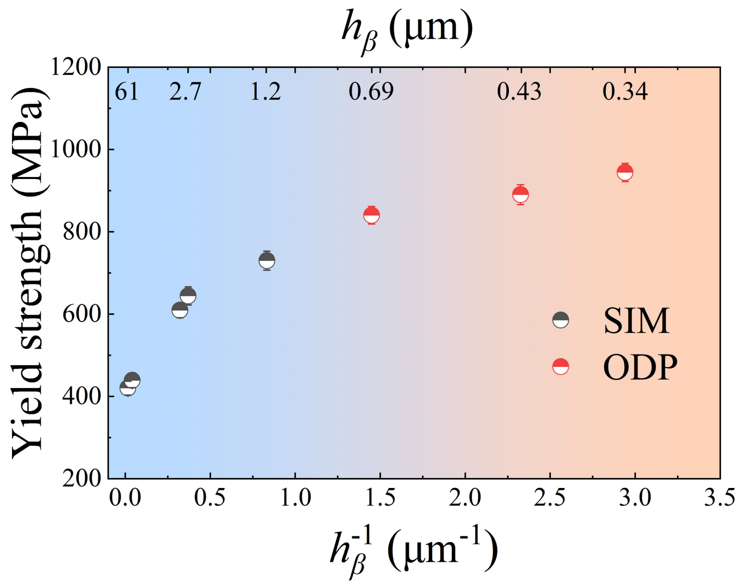

Supplementary Figure 12 The yield strength vs. inverse β-layer thickness of the present β-Ti alloys.

Supplementary Note 5. Plastic work density of the present β-Ti alloys

The plastic work density $W_n = \int_0^{\varepsilon_U} \sigma d\varepsilon$ is the work per unit volume dissipated until necking, sometimes called "toughness"[24]. The plastic work density is calculated by the direct integration of the true stress-strain curve data, and measured three times to obtain the average value.

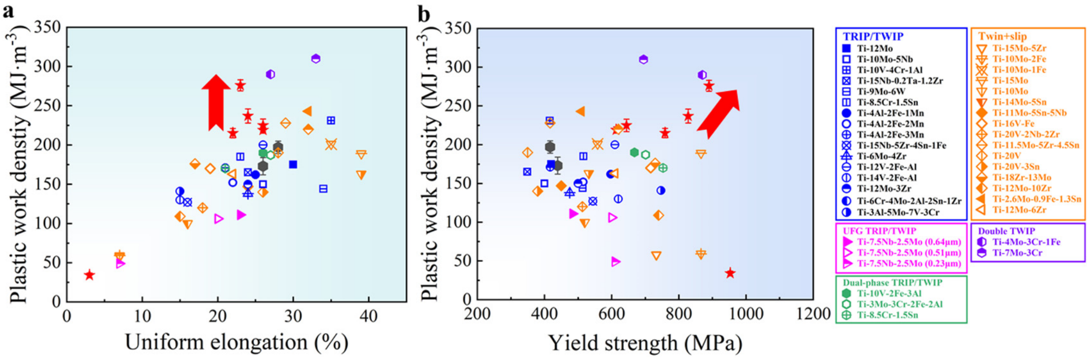

Supplementary Figure 13 The plastic work density of the present β-Ti alloys. Comparisons of **a** the plastic work density W_n *vs.* uniform elongation ε_U and **b** the plastic work density W_n *vs.* yield strength σ_y of the present and reported metastable β-Ti alloys, including TRIP/TWIP Ti alloys: Ti-12Mo[25], Ti-10Mo-5Nb[5], Ti-10V-4Cr-1Al[10], Ti-15Nb-0.2Ta-1.2Zr[26], Ti-9Mo-6W,[4] Ti-8.5Cr-1.5Sn[3], (Ti-4Al-2Fe-1Mn, Ti-4Al-2Fe-2Mn and Ti-4Al-2Fe-3Mn)[27], Ti-15Nb-5Zr-4Sn-1Fe[28], Ti-6Mo-4Zr[17], (Ti-12V-2Fe-1Al and Ti-14V-2Fe-1Al)[8], Ti-12Mo-3Zr[6] and Ti-6Cr-4Mo-2Al-2Sn-1Zr[29]; Dual-phase TRIP/TWIP Ti alloys: Ti-10V-2Fe-3Al[30], Ti-3Mo-3Cr-2Fe-2Al[31], Ti-8.5Cr-1.2Sn[32]; Twin+slip Ti alloys: Ti-3Al-5Mo-7V-3Cr[7], (Ti-15Mo-5Zr, Ti-10Mo-2Fe, Ti-10Mo-1Fe and Ti-15Mo) [18], Ti-10Mo, (Ti-14Mo-5Sn and Ti-11Mo-5Sn-5Nb)[5], Ti-16V-1Fe[8], Ti-20V-2Nb-2Zr[20], (Ti-11.5Mo-5Zr-4.5Sn, Ti-20V-3Sn and Ti-20V)[19], Ti-18Zr-13Mo[33], (Ti-12Mo-10Zr and Ti-12Mo-6Zr)[6], Ti-2.6Mo-0.9Fe-1.3Sn[34], UFG TRIP/TWIP Ti alloys: Ti-7.5Nb-2.5Mo (with different β grain sizes)[35]; Double TWIP Ti alloys: Ti-7Mo-3Cr[36] and Ti-4Mo-3Cr-1Fe[37] alloys. Error bars indicate standard deviations for three tests.

Supplementary Note 6. Fracture behavior and the underlying mechanism of EGS samples without and with α nanoprecipitates.

Here, to further support the role of the α nanoprecipitates, we characterized the fractography of the rupture surface of EGS samples without and with α precipitates, see Supplementary Fig. 14 and Supplementary Fig. 15, respectively.

Supplementary Fig. 14a-a1 show the dimple morphology on the center of the fracture surface of α nanoprecipitate-free EGS sample. It is found that the β grain boundaries (GBs) often serve as preferential sites for the nucleation of void, even of the initiation and propagation of microcracks owing to the stress concentration at the strain $\varepsilon = 0.33$ (Supplementary Fig. 14b1). Moreover, these microcrack can be blunted by these refined stress-induced martensites (SIMs), see white arrows in Supplementary Fig. 14b1, which can relieve stress concentrations[38]. At the strain of $\varepsilon = 0.36$, in addition to the observed voids and cracks at GBs, some voids initiate at martensite interfaces, see orange arrows in Supplementary Fig. 14b2. Also, some martensite variants are activated to blunt the crack tip, see white arrows in Supplementary Fig. 14b2. It is well known that these martensites could either enhance the strain hardening behavior via transformation-induced plasticity effect or serve as the nucleation sites of cracks by high stress concentrations[38]. Thus, at a strain ($\varepsilon = 0.40$) near the fracture strain, both voids and cracks are mainly concentrated at the α''/β phase interfaces, see Supplementary Fig. 14b3.

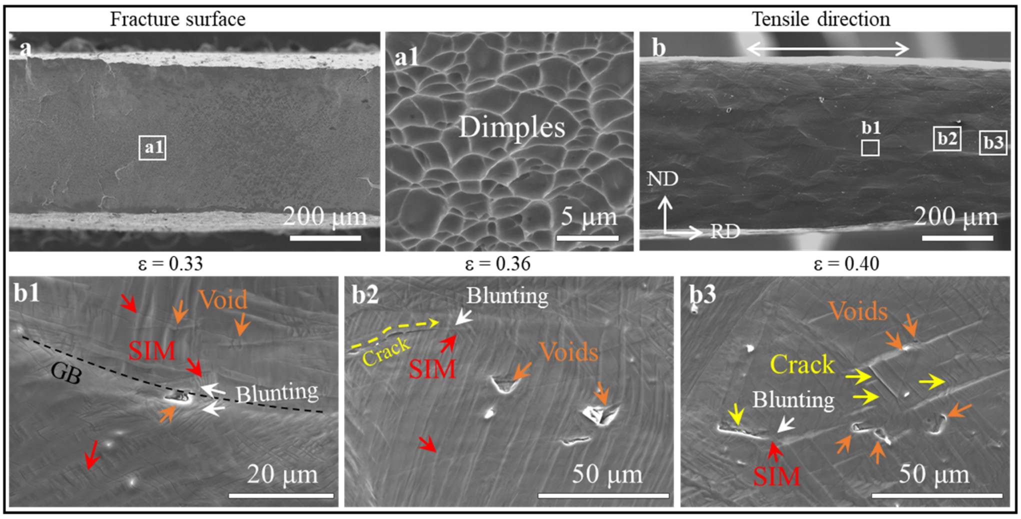

Supplementary Figure 14 Fracture behavior and the toughening mechanisms of EGS samples without α nanoprecipitates. **a** An SEM image of the fracture surface of EGS β-Ti alloys. **a1** A magnified image of the EGS fracture surface shows massive dimples, i.e., ductility fracture. **b** An SEM image of the post-fractured EGS specimen along RD. **b1** An SEM image shows void initiation at the β grain boundary at ε = 0.33, and cracks were blunted via dense stress-induced martensites (SIMs, white arrows). **b2** An SEM image shows voids initiation at the martensite interfaces at ε = 0.36, as marked via orange arrows. **b3** An SEM image shows crack initiation (orange arrows) and propagation (yellow arrows) at the martensite interfaces at ε = 0.40.

Next, the EGS samples with uniformly distributed α phases were stretched to fracture. SEM images and the corresponding inset show the dimple fracture for the EGS samples with α precipitates, see Supplementary Fig. 15a. Due to the existence of α particles, the incoherent α/β interfaces serve as preferential sites for the initiation and propagation of microcracks owing to stress concentrations caused by the strain incompatibility (orange arrows in Supplementary Fig. 15b1). Moreover, this stress concentration can trigger SIM nucleated from the α/β interface to release the local stress, as indicated by red arrows in Supplementary Fig. 15b1. On the other hand, the

α''/β interface is not sufficiently strong[38], so that the α''/β interfacial stress concentrations could cause severe deformation of martensites, promoting the initiation of voids, as indicated by yellow arrows in Supplementary Fig. 15b1. At a strain (ε = 0.41) near the fracture strain, densely refined SIM plates are severely deformed, and abundant microcracks nucleate to alleviate stress concentrations and delay the fracture via the well-known micro-crack toughening mechanism, as shown in Supplementary Fig. 15b2. Although cracks tend to propagate along the martensitic lath during deformation (similar to the case of α nanoprecipitate-free EGS alloys in Fig. Supplementary Fig. 14b3), there is a deflected cracking path as they approach α particles (white arrows in Supplementary Fig. 15b2), which supports the role of the α nanoprecipitates in delaying fracture. Therefore, based on above findings, we claim that these α nanoprecipitates play important roles in ductilizing and toughening the present Ti alloys, as supported by the mechanical responses in Supplementary Fig. 16.

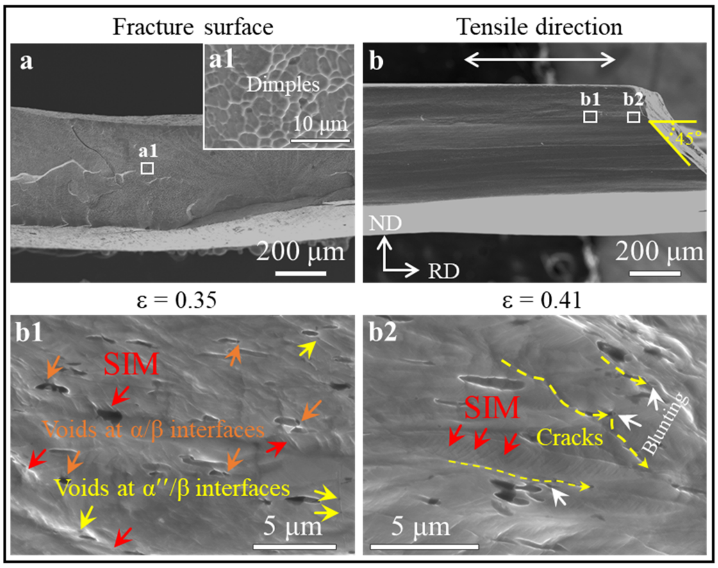

Supplementary Figure 15 Fracture behavior and the underlying toughening mechanism of EGS β-Ti alloys containing α nanoprecipitates. **a** An SEM image of the fracture surface of EGS with α nanoprecipitates β-Ti alloys. The inset shows massive

dimples, i.e., ductility fracture. **b** An SEM image of the post-fractured EGS with α nanoprecipitates specimen along RD. **b1** An SEM image shows void initiation at α/β interfaces and α″/β phase boundaries (PBs) at ε = 0.35, and voids were blunted via refined SIMs (red arrows). **b2** An SEM image shows cracks are deflected and blunted as they approach α particles (white arrows) at ε = 0.41.

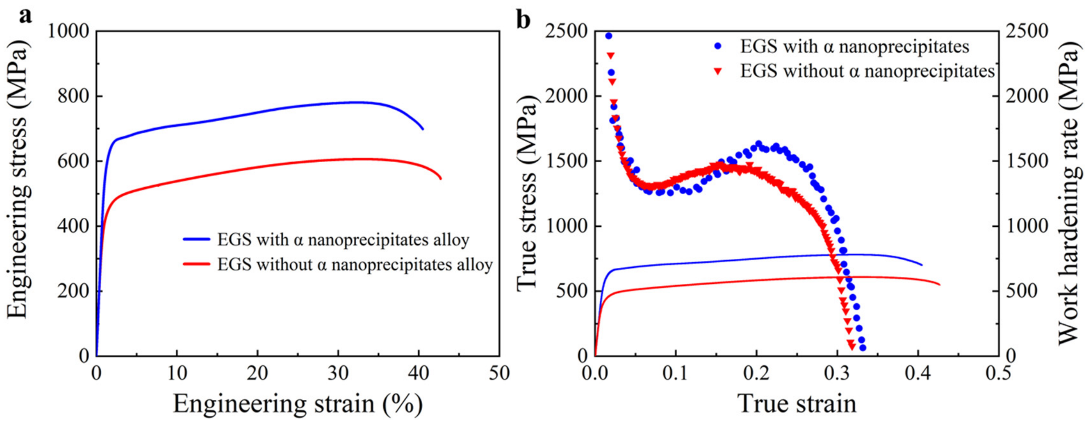

Supplementary Figure 16 Mechanical responses of EGS samples without and with α nanoprecipitates. **a** Engineering stress-strain curves for the alloys at different conditions. **b** The corresponding strain-hardening response. Intriguingly, the EGS sample with α nanoprecipitates shows significant hardening ability, implying α nanoprecipitates can ductilize and toughen the present (EGS) alloys.

Supplementary Note 7. Nanoindentation testing for the strength discrepancy between α and β phases

The hardness of the β matrix and α/β regions was measured using a TI950 TriboIndenter (Hysitron, Minneapolis, MN) with a standard Berkovich tip at room temperature, following the Oliver-Pharr method adopted in our previous work[39-41]. Indeed, it is found that α nanoprecipitates are harder than the β-Ti matrix (see Supplementary Fig. 17 for HLS-0.43 alloys), i.e., harder nanoprecipitates can serve as local microscopic stress raisers to enhance the TRIP of the soft β-matrix[13].

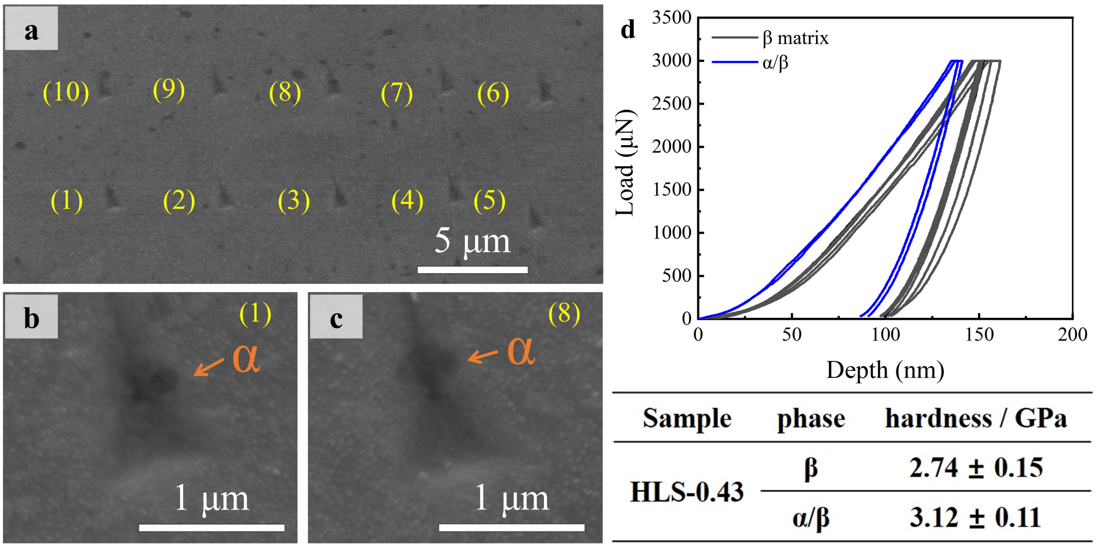

Sample	phase	hardness / GPa
HLS-0.43	β	2.74 ± 0.15
	α/β	3.12 ± 0.11

Supplementary Figure 17 Nanoindentation hardness measurements for the HLS-0.43 β-Ti alloys. **a** An SEM image showing the region of interest with a 2 × 5 indent grid. **b-c** The indents located in the α/β regions are displayed via orange arrows. **d** The load-displacement curves of both the β and the α/β regions.

Supplementary Note 8. Evolution of the microcrack density during tension in HLS-0.43 β-Ti alloys

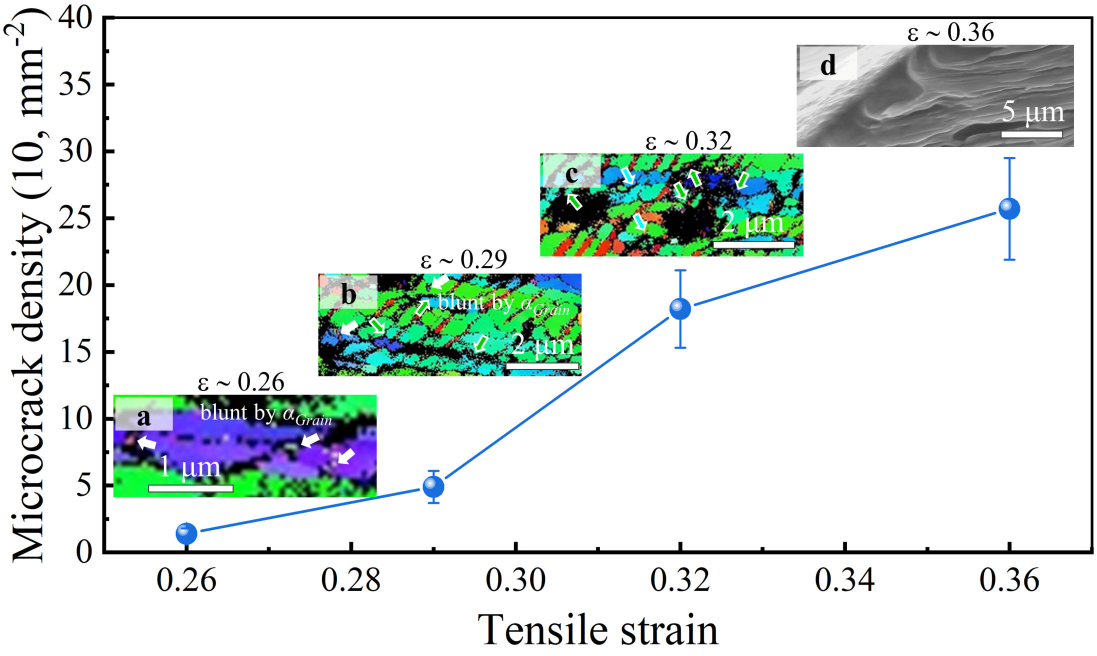

Supplementary Figure 18 Evolution of the microcrack density during tension in HLS-0.43 β-Ti alloys. The insets show the typical cracking features at four plastic strains ε of 0.26, 0.29, 0.32 and 0.36, respectively. **a** A typical EBSD image showing a crack initiation and propagation along the α/β interfaces at ε ~ 0.26, and cracks were blunted by α_{Grain} (white arrows). **b** A typical EBSD image showing crack deflection caused by layer interfaces (green arrows) and α_{Grain} (white arrow) at ε ~ 0.29. **c** A typical EBSD image showing crack deflection (green arrows) and branch (light blue arrows) at ε ~ 0.32. **d** Interface delamination at ε~ 0.36. Error bars indicate standard deviations for three statistics.

Supplementary Note 9. Theoretical calculations of strengthening responses

For the coarse-grained EGS samples (see Supplementary Fig. 19), in addition to the lattice resistance for gliding dislocations (σ_o), grain boundary (GB) strengthening (σ_{HP}) and dislocation strengthening (σ_ρ) were considered, thus the yield strength (σ_y) can be expressed as:

$$\sigma_y = \sigma_o + \sigma_\rho + \sigma_{HP} \quad (3)$$

where σ_o is the lattice friction stress for dislocations (including the solid solution strengthening). The dislocation hardening, σ_ρ, is described via the Taylor model [42]:

$$\sigma_\rho = \alpha M \mu b \sqrt{\rho} \quad (4)$$

where α is a constant ~0.3, μ = 39 GPa is the shear modulus, M = 2.8 is the Taylor factor, b = 2.8 Å is the Burgers vector (refer to Ref.[43] for all parameters), and ρ is the dislocation density, on the order of 7.3 ~ 7.5 × 10^{13} m^{-2} for EGS β-Ti alloys[43]. The GB strengthening can be evaluated via the Hall-Petch (H-P) relationship[44,45]:

$$\sigma_{HP} = Kd^{-1/2} \quad (5)$$

where d is the average grain size, and K is the H-P slope. Taking the parameters mentioned above, we can simply obtain the lattice friction stress of σ_o ~275 MPa for the present EGS β-Ti alloys, as inferred from the intercept of the H-P relationship between yield strength and the inverse square of grain sizes.

For these HLS samples, apart from GB strengthening and dislocation strengthening, the strength contribution from both intragranular (σ_{intra}) and intergranular (σ_{inter}) hard α nanoprecipitates can be estimated:

$$\sigma_y = \sigma_{Matrix} + \sigma_{intra} + \sigma_{inter} = (\sigma_o + \sigma_\rho + \sigma_{HP}) + \sigma_{intra} + \sigma_{inter} \quad (6)$$

Given the intragranular nanoparticles can hind dislocations moving in the grain

interior, σ_{intra} is thus given by[46]:

$$\sigma_{intra} = M\frac{0.4\mu b}{\pi\sqrt{1-\nu}}\frac{\ln(\frac{2r}{b})}{\lambda_{intra}} \tag{7}$$

where M = 2.8 is the Taylor factor, μ~39 GPa is the shear modulus of the BCC matrix[43], b (= 2.8 Å) is the Burgers vector[47], ν = 0.32 is the Poisson ratio[47], and r is the average radius of the precipitates. d_{intra}, λ_{intra}, and f_{intra} are the particle size, the interparticle spacing, and the volume fraction of α_{Grain} particles, respectively, as shown in Supplementary Fig. 20. Note that individual grains rarely contain two α_{Grain} particles, and most of them contain only one α_{Grain} in HLS alloys, see Supplementary Fig. 20. Therefore, we use $\lambda_{intra} = 2r(\sqrt{\frac{\pi}{4f_{intra}}} - 1)$[48,49] to describe the interparticle spacing of sphere-like α_{Grain} with an aspect ratio of ~1. Thus, the strength contribution of intragranular (σ_{intra}) α precipitates is ~41 MPa for HLS-0.43 Ti alloys.

Moreover, the strength contribution from intergranular α precipitates, including spherical α_{GB} and fibrous α_{Int} in our HLS β-Ti alloys, can be calculated as follows:

$$\sigma_{inter} = \sigma_{inter}^{GB} + \sigma_{inter}^{Int} \tag{8}$$

For the intergranular sphere-like α_{GB} particles, they are located at GBs without significant influences on dislocation motion, and thus their contribution to the strength (σ_{inter}^{GB}) is caused by the load-bearing effect[50]. In this regard, a simple but generally adopted expression for the load-bearing strength was employed[49,51]:

$$\sigma_{inter}^{GB} = 0.5 f_{\alpha GB}\sigma_{Matrix} \tag{9}$$

where $f_{\alpha GB}$ is the volume fraction of intergranular particles and σ_{Matrix} the yield strength of the matrix[49]. The strength contribution of intergranular α_{GB} precipitates is σ_{inter}^{GB} ~9 MPa for HLS-0.43 Ti alloys.

According to the work of Kelly and Davis[52], the strengthening caused by the

fiber-like α_{Int} (σ_{inter}^{Int}) depends on their aspect ratios and volume fractions. Therefore, for the intergranular fiber-like α_{Int} precipitates, the fiber-reinforced expression is usually given by[53]:

$$\sigma_{inter}^{int} = f_{\alpha Int}\left(\sigma_{Matrix} + \frac{8 f_{\alpha Int}(E_{\alpha} - E_{matrix})\gamma^2}{3(E_{\alpha} + 4\gamma^2 f_{\alpha Int} E_{matrix})}\sigma_{Matrix}\right) \quad (10)$$

where γ is the aspect ratio, E_{α} is the Young's modulus of the α phase ~114 GPa[54], $f_{\alpha Int}$ is the volume fraction of α_{Int} precipitates, and E_{matrix} is the Young's modulus of the BCC matrix (Based on the tensile curves of EGS-24 and EGS-61 samples in Supplementary Fig. 19, the Young's modulus of the BCC matrix is approximately 51 GPa). Taking above parameters and those listed in Supplementary Table 2, the strength contribution of fiber-like α_{Int} precipitates is σ_{inter}^{Int} ~10.1 MPa for the HLS-0.43 alloys.

When the grain size is in the micron-scale, i.e., EGS, HLS-3.2, HLS-2.7 and HLS-1.2 β-Ti alloys, taking the H-P slope K_{SIM} = 280 MPa·μm$^{1/2}$, the calculated strength agrees well with the experimental data. As the β layer/grain size is reduced to the submicron-scale, taking K_{ODP} = 240 MPa·μm$^{1/2}$, the calculated strength also agrees well with the experimental results, as shown in Supplementary Fig. 19b.

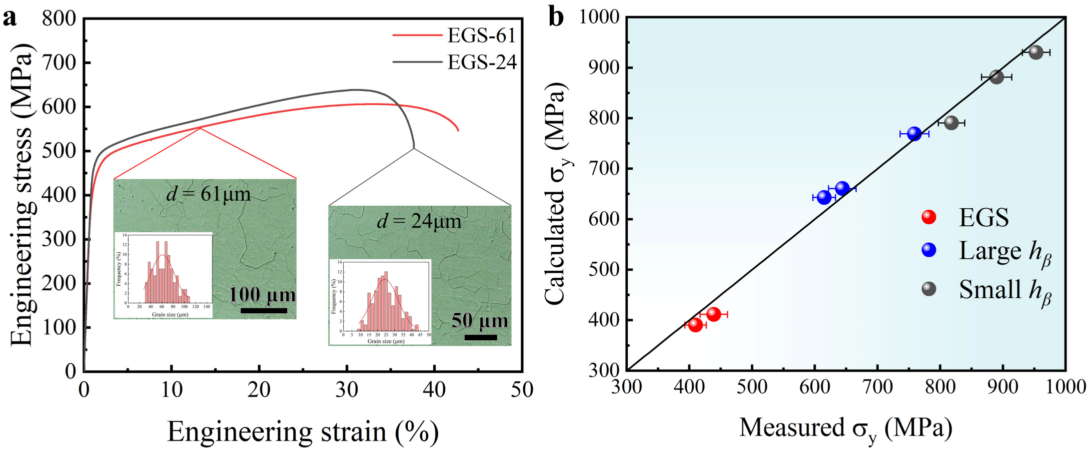

Supplementary Figure 19 Mechanical responses of the present EGS β-Ti alloys and

a comparison of the measured and calculated yield strength. **a** The engineering stress-strain curves for the present two EGS β-Ti alloys, the average grain sizes are 61 μm and 24 μm, respectively. Based on the mechanical properties and grain sizes of EGS-61 and EGS-24 β-Ti alloys, we deduced that the lattice friction stress is ~275 MPa. **b** A comparison between the calculated and the experimentally measured yield strength of EGS and HLS β-Ti alloys. Error bars indicate standard deviations.

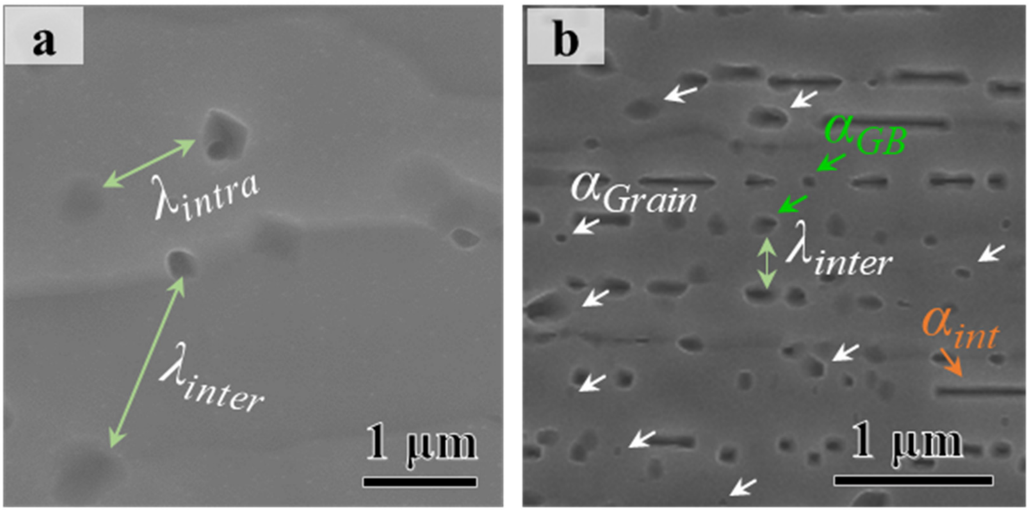

Supplementary Figure 20 The definition of the spacing of intragranular and intragranular particles in HLS β-Ti alloys. **a** The spacing of intragranular α_{Grain} and intragranular α particles (including α_{GB} and α_{Int}) in HLS-1.2 alloys. **b** Each β-grain contains only one α_{Grain} particle in the HLS-0.43 alloys.

Supplementary Note 10. Summary of the mechanical properties of the present and other β-Ti alloys

Supplementary Table 4 Summary of the mechanical properties of the present β-Ti alloys.

Material	Yield strength (MPa)	Ultimate tensile strength (MPa)	Uniform elongation (%)	Plastic work density (MJ·m^{-3})	Fracture work density (MJ·m^{-3})
HLS-0.34	953 ± 19	1001 ± 17	3 ± 1.0	34 ± 3	74 ± 5
HLS-0.43	890 ± 14	975 ± 11	23 ± 1.1	276 ± 7	350 ± 8
HLS-0.69	828 ± 16	923 ± 14	24 ± 1.2	237 ± 9	324 ± 7
HLS-1.2	759 ± 17	896 ± 16	22 ± 1.1	215 ± 6	310 ± 7
HLS-2.7	644 ± 13	825 ± 10	26 ± 1.3	225 ± 8	321 ± 6
HLS-3.2	613 ± 15	794 ± 11	26 ± 1.0	219 ± 4	303 ± 5
EGS-24	439 ± 18	638 ± 10	26 ± 1.5	173 ± 11	217 ± 10
EGS-61	417 ± 14	605 ± 12	28 ± 1.4	197 ± 8	239 ± 6

Supplementary Table 5 Compositions and mechanical properties of the β-Ti alloys reported previously.

Alloys	Yield strength (MPa)	Uniform elongation	Total elongation (%)	Ref.
Ti-3Al-5Mo-7V-3Cr	747	15%	21	7
Ti-15Mo-5Zr	733	7%	18	18
Ti-10Mo-2Fe	866	7%	17	
Ti-10Mo-1Fe	558	35%	42	55
Ti-15Mo	434	39%	48	
Ti-14Mo-5Sn	520	16%	19	5
Ti-11Mo-5Sn-5Nb	450	24%	26	
Ti-16V-Fe	716	19%	30	8
Ti-20V-2Nb-2Zr	531	18%	32	20
Ti-11.5Mo-5Zr-4.5Sn	471	29%	33	19
Ti-20V	358	28%	34	
Ti-6Cr-4Mo-2Al-2Sn-1Zr	620	32%	41	29
Ti-20V-3Sn	380	26%	32	19
Ti-18Zr-13Mo	780	17%	24	33
Ti-2.6Mo-0.9Fe-1.3Sn	740	15%	18	34
Ti-12Mo-6Zr	510	32%	39	6
Ti-12Mo-10Zr	610	22%	29	
Ti-10Mo	532	39%	49	56
Ti-12Mo	494	30%	44	25

Ti-10Mo-5Nb	400	26%	33	5
Ti-10V-4Cr-1Al	416	34%	35	10
Ti-15Nb-0.2Ta-1.2Zr	349	24%	30	26
Ti-9Mo-6W	514	34%	34	4
Ti-8.5Cr-1.5Sn	516	23%	36	3
Ti-4Al-2Fe-1Mn	597	25%	34	
Ti-4Al-2Fe-2Mn	515	22%	26	27
Ti-4Al-2Fe-3Mn	417	21%	31	
Ti-15Nb-5Zr-4Sn-1Fe	544	16%	20	28
Ti-6Mo-4Zr	475	24%	27	17
Ti-12V-2Fe-Al	610	26%	39	8
Ti-14V-2Fe-Al	620	15%	31	
Ti-12Mo-3Zr	500	24%	36	6
Ti-10Cr	728	13%	14	
Ti-11Cr	803	14%	16	57
Ti-12Cr	748	12%	16	
Ti-30Zr-4Cr	496	9%	24	
Ti-30Zr-2Cr-5Mo	511	23%	24	58
Ti-30Zr-2Cr-4Mo	596	26%	26	
Ti-30Zr-2Cr-3Mo	648	12%	29	
Ti-7.5Nb-2.5Mo (0.64μm)	484	23%	46	
Ti-7.5Nb-2.5Mo (0.51μm)	600	20%	47	35
Ti-7.5Nb-2.5Mo (0.23μm)	610	7%	26	
Ti-10V-2Fe-3Al (dual phase)	668	26%	36	30
Ti-3Mo-3Cr-2Fe-2Al (dual phase)	701	27%	40	31
Ti-8.5Cr-1.5Sn (dual phase)	753	21%	33	59
Ti-7Mo-3Cr	695	33%	47	36
Ti-4Mo-3Cr-1Fe	870	27%	41	37

Supplementary references

1. Kuroda D, Niinomi M, Morinaga M, Kato Y, Yashiro T. Design and mechanical properties of new β type titanium alloys for implant materials. *Mater Sci Eng A* **243**, 244-249 (1998).
2. Marteleur M, et al. On the design of new β-metastable titanium alloys with improved work hardening rate thanks to simultaneous TRIP and TWIP effects. *Scr Mater* **66**, 749-752 (2012).
3. Brozek C, et al. A β-titanium alloy with extra high strain-hardening rate: Design and mechanical properties. *Scr Mater* **114**, 60-64 (2016).
4. Sun F, et al. A new titanium alloy with a combination of high strength, high strain hardening and improved ductility. *Scr Mater* **94**, 17-20 (2015).
5. Zhao G, Xu X, Dye D, Rivera-Díaz-del-Castillo P E J, Petrinic N. Facile route to implement transformation strengthening in titanium alloys. *Scr Mater* **208**, 114362 (2022).
6. Qian B, et al. In-situ microstructural investigations of the TRIP-to-TWIP evolution in Ti-Mo-Zr alloys as a function of Zr concentration. *J Mater Sci Technol* **65**, 228-237 (2021).
7. Sadeghpour S, et al. A new multi-element beta titanium alloy with a high yield strength exhibiting transformation and twinning induced plasticity effects. *Scr Mater* **145**, 104-108 (2018).
8. Wang W, Zhang X, Sun J. Phase stability and tensile behavior of metastable β Ti-V-Fe and Ti-V-Fe-Al alloys. *Mater Charact* **142**, 398-405 (2018).
9. Bignon M, Bertrand E, Tancret F, Rivera-Díaz-del-Castillo P E J. Modelling martensitic transformation in titanium alloys: The influence of temperature and deformation. *Materialia* **7**, 100382 (2019).
10. Lilensten L, et al. On the heterogeneous nature of deformation in a strain-transformable beta metastable Ti-V-Cr-Al alloy. *Acta Mater* **162**, 268-276 (2019).
11. Lütjering G, Williams J C. *Titanium*. Springer Science & Business Media (2007).
12. Zhang T, et al. A new α + β Ti-alloy with refined microstructures and enhanced mechanical properties in the as-cast state. *Scr Mater* **207**, 114260 (2022).
13. Choudhuri D, et al. Enhancing strength and strain hardenability via deformation twinning in fcc-based high entropy alloys reinforced with intermetallic compounds. *Acta Mater* **165**, 420-430 (2019).
14. Banerjee D, Williams J C. Perspectives on Titanium Science and Technology. *Acta Mater* **61**, 844-879 (2013).
15. Abdel-Hady M, Hinoshita K, Morinaga M. General approach to phase stability and elastic properties of β-type Ti-alloys using electronic parameters. *Scr Mater* **55**, 477-480 (2006).
16. Hémery S, Villechaise P, Banerjee D. Microplasticity at Room Temperature in α/β Titanium Alloys. *Metall Mater Trans A* **51**, 4931–4969 (2020).
17. Wang C H, Russell A M, Cao G H. A semi-empirical approach to the prediction of deformation behaviors of β-Ti alloys. *Scr Mater* **158**, 62-65 (2019).
18. Min X H, Tsuzaki K, Emura S, Tsuchiya K. Enhancement of uniform elongation in high strength Ti–Mo based alloys by combination of deformation modes. *Mater Sci Eng A* **528**, 4569-4578 (2011).
19. Hanada S, Izumi O. Correlation of tensile properties, deformation modes, and phase stability in commercial β-phase titanium alloys. *Metall Mater Trans A* **18**, 265-271 (1987).
20. Wang W L, Wang X L, Mei W, Sun J. Role of grain size in tensile behavior in twinning-induced plasticity β Ti-20V-2Nb-2Zr alloy. *Mater Charact* **120**, 263-267 (2016).

21. Chen W, et al. Origin of the ductile-to-brittle transition of metastable β-titanium alloys: Self-hardening of ω-precipitates. *Acta Mater* **170**, 187-204 (2019).
22. Jang T J, et al. Shear band-driven precipitate dispersion for ultrastrong ductile medium-entropy alloys. *Nat Commun* **12**, 4703 (2021).
23. Mao Q, Zhang Y, Liu J, Zhao Y. Breaking Material Property Trade-offs via Macrodesign of Microstructure. *Nano Lett* **21**, 3191-3197 (2021).
24. Niu G, et al. Superior fracture toughness in a high-strength austenitic steel with heterogeneous lamellar microstructure. *Acta Mater* **226**, 117642 (2022).
25. Choisez L, Elmahdy A, Verleysen P, Jacques P J. Fracture mechanisms in flat and cylindrical tensile specimens of TRIP-TWIP β-metastable Ti-12Mo alloy. *Acta Mater* **220**, 117294 (2021).
26. Lai M J, Li T, Raabe D. ω phase acts as a switch between dislocation channeling and joint twinning- and transformation-induced plasticity in a metastable β titanium alloy. *Acta Mater* **151**, 67-77 (2018).
27. Oh J M, et al. High strength and ductility in low-cost Ti–Al–Fe–Mn alloy exhibiting transformation-induced plasticity. *Mater Sci Eng A* **772**, 138813 (2020).
28. Fu Y, et al. Ultrahigh strain hardening in a transformation-induced plasticity and twinning-induced plasticity titanium alloy. *Scr Mater* **187**, 285-290 (2020).
29. Ren L, Xiao W, Ma C, Zheng R, Zhou L. Development of a high strength and high ductility near β-Ti alloy with twinning induced plasticity effect. *Scr Mater* **156**, 47-50 (2018).
30. Danard Y, et al. Microstructure design and in-situ investigation of TRIP/TWIP effects in a forged dual-phase Ti–10V–2Fe–3Al alloy. *Materialia* **8**, 100507 (2019).
31. Lee S W, Park C H, Hong J-K, Yeom J-T. Development of sub-grained α+β Ti alloy with high yield strength showing twinning- and transformation-induced plasticity. *J Alloys Compd* **813**, 152102 (2020).
32. Villechaise P, et al. Design and development of a dual-phase TRIP-TWIP alloy for enhanced mechanical properties. *MATEC Web of Conferences* **321**, 11014 (2020).
33. Zhang J, et al. Strong and ductile beta Ti–18Zr–13Mo alloy with multimodal twinning. *Mater Res Lett* **7**, 251-257 (2019).
34. Xu Y, Gao J, Huang Y, Rainforth W M. A low-cost metastable beta Ti alloy with high elastic admissible strain and enhanced ductility for orthopaedic application. *J Alloys Compd* **835**, 155391 (2020).
35. Zhang B, et al. Achieving large super-elasticity through changing relative easiness of deformation modes in Ti-Nb-Mo alloy by ultra-grain refinement. *Mater Res Lett* **9**, 223-230 (2021).
36. Gao J, et al. Deformation mechanisms in a metastable beta titanium twinning induced plasticity alloy with high yield strength and high strain hardening rate. *Acta Mater* **152**, 301-314 (2018).
37. Ren L, et al. Simultaneously enhanced strength and ductility in a metastable β-Ti alloy by stress-induced hierarchical twin structure. *Scr Mater* **184**, 6-11 (2020).
38. Xiao J F, Shang X K, Hou J H, Li Y, He B B. Role of stress-induced martensite on damage behavior in a metastable titanium alloy. *Int J Plast* **146**, 103103 (2021).
39. Niu J J, et al. Size-dependent deformation mechanisms and strain-rate sensitivity in nanostructured Cu/X (X=Cr, Zr) multilayer films. *Acta Mater* **60**, 3677-3689 (2012).
40. Zhao J T, et al. Zr alloying effect on the microstructure evolution and plastic deformation of nanostructured Cu thin films. *Acta Mater* **132**, 550-564 (2017).
41. Zhang J Y, et al. Alloying effects on the microstructure and mechanical properties of

nanocrystalline Cu-based alloyed thin films: Miscible Cu-Ti vs immiscible Cu-Mo. *Acta Mater* **143**, 55-66 (2018).

42. Kocks U F, Mecking H. Physics and phenomenology of strain hardening: the FCC case. *Prog Mater Sci* **48**, 171-273 (2003).
43. Zhao G-H, Xu X, Dye D, Rivera-Díaz-del-Castillo P E J. Microstructural evolution and strain-hardening in TWIP Ti alloys. *Acta Mater* **183**, 155-164 (2020).
44. El-Awady J A. Unravelling the physics of size-dependent dislocation-mediated plasticity. *Nat Commun* **6**, 5926 (2015).
45. Pande C S, Cooper K P. Nanomechanics of Hall–Petch relationship in nanocrystalline materials. *Prog Mater Sci* **54**, 689-706 (2009).
46. Ardell A J. Precipitation hardening. *Metallurgical Transactions A* **16**, 2131-2165 (1985).
47. Zhao G H, Liang X Z, Kim B, Rivera-Díaz-del-Castillo P E J. Modelling strengthening mechanisms in beta-type Ti alloys. *Mater Sci Eng A* **756**, 156-160 (2019).
48. Zhu Z, et al. Superior mechanical properties of a selective-laser-melted AlZnMgCuScZr alloy enabled by a tunable hierarchical microstructure and dual-nanoprecipitation. *Mater Today* **52**, 90-101 (2021).
49. Liu G, et al. Nanostructured high-strength molybdenum alloys with unprecedented tensile ductility. *Nat Mater* **12**, 344-350 (2013).
50. Scudino S, Liu G, Sakaliyska M, Surreddi K B, Eckert J. Powder metallurgy of Al-based metal matrix composites reinforced with β-Al3Mg2 intermetallic particles: Analysis and modeling of mechanical properties. *Acta Mater* **57**, 4529-4538 (2009).
51. Starink M J, Syngellakis S. Shear lag models for discontinuous composites: fibre end stresses and weak interface layers. *Mater Sci Eng A* **270**, 270-277 (1999).
52. Kelly A, Davies G. The principles of the fibre reinforcement of metals. *Metallurgical Reviews* **10**, 1-77 (1965).
53. Nardone V C, Prewo K M. On the strength of discontinuous silicon carbide reinforced aluminum composites. *Scripta Metallurgica* **20**, 43-48 (1986).
54. de Formanoir C, et al. Micromechanical behavior and thermal stability of a dual-phase α+α' titanium alloy produced by additive manufacturing. *Acta Mater* **162**, 149-162 (2019).
55. Min X H, et al. Effects of Fe addition on tensile deformation mode and crevice corrosion resistance in Ti–15Mo alloy. *Mater Sci Eng A* **527**, 2693-2701 (2010).
56. Min X H, Emura S, Nishimura T, Tsuchiya K, Tsuzaki K. Microstructure, tensile deformation mode and crevice corrosion resistance in Ti–10Mo–xFe alloys. *Mater Sci Eng A* **527**, 5499-5506 (2010).
57. Zhao X, et al. Optimization of Cr content of metastable beta-type Ti-Cr alloys with changeable Young's modulus for spinal fixation applications. *Acta Biomater* **8**, 2392-2400 (2012).
58. Zhao X, Niinomi M, Nakai M, Miyamoto G, Furuhara T. Microstructures and mechanical properties of metastable Ti-30Zr-(Cr, Mo) alloys with changeable Young's modulus for spinal fixation applications. *Acta Biomater* **7**, 3230-3236 (2011).
59. Lilensten L, et al. From single phase to dual-phase TRIP-TWIP titanium alloys: Design approach and properties. *Materialia* **12**, 100700 (2020).
